# Catalytic and Antitubercular Activities of Multifunctional Copper(II)–Schiff Base Complexes: Insights into Structure, Theoretical Calculations, and Physicochemical Behavior

**DOI:** 10.1021/acs.inorgchem.5c05547

**Published:** 2026-05-20

**Authors:** Anna Jurowska, Weronika Bogdał, Janusz Szklarzewicz, Mateusz Brela, Maciej Hodorowicz, Patrycja Miller, Mateusz Janeta, Agnieszka Głogowska, Ewa Augustynowicz-Kopeć, Ghodrat Mahmoudi

**Affiliations:** † Faculty of Chemistry, Jagiellonian University, Gronostajowa 2, Kraków 30-387, Poland; ‡ Faculty of Chemistry, 49572 University of Wrocław, F. Joliot-Curie 14, Wrocław 50-383, Poland; § Department of Microbiology, National Tuberculosis and Lung Diseases Research Institute, Warsaw 01-138, Poland; ∥ Department of Chemistry, Faculty of Science, University of Maragheh, P.O. Box 55181-83111, Maragheh 97HF+498, Iran; ⊥ Department of Chemistry, Dogus University, Dudullu-Ümraniye, Istanbul 34775, Turkey

## Abstract

Three copper­(II) complexes featuring tridentate ONO donor Schiff base ligands were synthesized and thoroughly characterized by X-ray crystallography, spectroscopy, and electrochemical methods. Density functional theory (DFT) calculations provided insights into their electronic structures and ligand–metal interactions. Complex **1** exhibited moderate catalytic activity toward the selective oxidation of toluene and styrene under mild conditions. Biological assays demonstrated measurable antimycobacterial effects against drug-sensitive and -resistant *Mycobacterium tuberculosis* strains, while combination studies with standard anti-TB drugs revealed mainly additive or indifferent interactions. These results highlight the promise of these copper­(II) Schiff-base complexes as multifunctional agents with catalytic and therapeutic applications.

## Graphical abstract

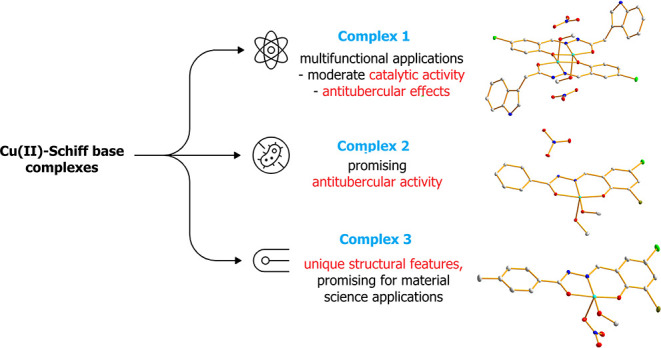

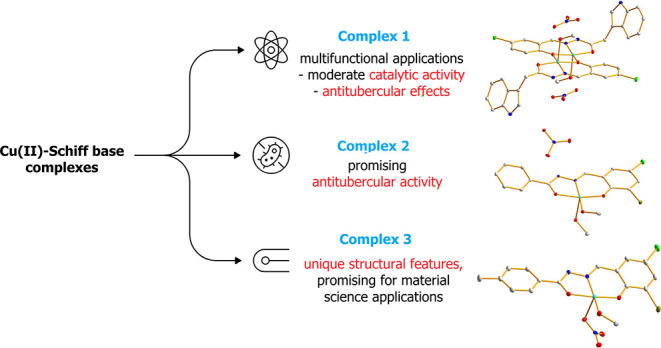

## Introduction

In recent years, Schiff bases have been extensively used as ligands in the synthesis of advanced molecular materials due to their wide range of applications and interesting biological and catalytic properties, especially with copper­(II) as a metal center.^1–8^ Their main advantages include ease and short synthesis time, as well as a wide range of modification options, for example, by introducing substituents into the aromatic rings of the components they comprise. In our study, this included the modification of salicylic aldehydes and benzhydrazides. Research on this group of compounds, and particularly copper­(II) complexes with such ligands, is particularly interesting due to the potential for obtaining various structures – monomers, dimers, polymers, and both ionic and neutral compounds.^9–12^ Looking from the point of view of the structure of these compounds, a question arises: what determines the formation of a given type of compound? Is the synthesis method important (factors such as temperature, time, and crystallization process)? The answer to this question is crucial because it allows for the possibility of directing the reaction toward the desired product for a given purpose.

Selective oxidation of aromatic hydrocarbons remains a pivotal transformation in organic synthesis, producing valuable intermediates such as benzaldehyde, benzoic acid, and styrene oxide.^13^ Copper-based catalysts have emerged as effective and environmentally benign catalysts for these transformations due to their redox versatility and low cost. In toluene oxidation, copper catalysts activate benzylic C–H bonds to yield benzyl alcohol, which undergoes further oxidation to benzaldehyde and benzoic acid. The oxidation of styrene is more complex, proceeding via potential pathways including epoxidation, allylic oxidation, and oxidative cleavage. Copper­(II) complexes have demonstrated the capacity to direct these pathways selectively, while supported copper nanoparticles have shown efficacy in promoting allylic oxidation to benzaldehyde.^14,15^

Beyond catalysis, copper­(II) Schiff base complexes have exhibited promising biological activities, including antimicrobial and antitubercular effects, addressing the urgent need for new therapeutics against drug-resistant bacterial strains. Tuberculosis (TB) is an infectious disease that remains a significant global health problem. According to the World Health Organization (WHO), in 2023, there were 10.8 million TB cases, and 1.25 million people died of this entirely curable disease. Data show that, after the COVID-19 pandemic, TB again became the leading cause of global mortality due to a single infectious agent and caused almost twice as many deaths as HIV/AIDS.^16^

The standard treatment for drug-susceptible TB is a six-month regimen combining isoniazid, rifampicin, pyrazinamide, and ethambutol.^17^ While this is successful for most patients, the lengthy duration can lead to poor compliance and encourage the development of drug-resistant strains. Drug-resistant TB presents formidable challenges as it requires the administration of second-line drugs that are less effective, more toxic, and more expensive.^18^

Despite the recent approval of new agents such as bedaquiline, delamanid, and pretomanid,^19^ the range of available therapies remains insufficient to halt the global spread of drug-resistant TB.^16,20^ Therefore, there is an urgent need for innovative chemotherapeutics with novel mechanisms of action. One promising area of research is the exploration of metal-containing organic compounds, which may offer unique ways to overcome resistance and improve treatment outcomes.

In this study, we report the synthesis, structural characterization, theoretical calculations, catalytic evaluation, and antitubercular potential of novel copper­(II) complexes with tridentate ONO donor Schiff base ligands. These complexes demonstrate distinctive structural features, effective catalytic oxidation of aromatic substrates, and promising activity against drug-sensitive and drug-resistant *Mycobacterium tuberculosis* strains, highlighting their potential as multifunctional molecular materials.

## Experimental Section

Cu­(NO_3_)_2_·3H_2_O, 5-chlorosalicylaldehyde, 3-bromo-5-chloro-salicylaldehyde, indole 3-acetic hydrazide, p-toluic hydrazide, benzhydrazide, 5.5 M *tert*-butyl hydroperoxide (tBuOOH) in dodecane, toluene, chlorobenzene, mesitylene, styrene, oleic acid-albumin-dextrose-catalase, Tween 80, isoniazid, rifampicin, ethambutol were of analytical grade and were used as supplied. All solvents were of analytical grade and were used as supplied. BaSO_4_ was of spectroscopic grade (Shimadzu, Japan). Microanalysis of carbon, hydrogen, and nitrogen was performed using an Elementar Vario MICRO Cube elemental analyzer. Electronic absorption spectra were recorded with a Shimadzu UV-3600 UV–vis-NIR spectrophotometer equipped with a CPS-240 temperature controller. Diffuse reflectance spectra were measured in BaSO_4_ pellets, with BaSO_4_ as a reference, on a Shimadzu UV-3600 UV–vis-NIR equipped with an ISR-240 integrating sphere attachment in the 200–1000 nm range. IR spectra were recorded on a Nicolet iS5 FT-IR spectrophotometer. The magnetic susceptibility measurement was performed on a Sherwood Scientific magnetic susceptibility balance. CV measurements were carried out in dimethyl sulfoxide (DMSO) with [Bu_4_N]­PF_6_ (0.1 M) as supporting electrolyte, using Pt working and counter and Ag/AgCl reference electrodes on an Autolab PGSTAT128N potentiostat/galvanostat. *E*_1/2_ values were calculated from the average anodic and cathodic peak potentials as *E*_1/2_ = 0.5­(*E*_a_ + *E*_c_). The oxidation products were determined and analyzed by gas chromatography. GC–MS (EI) data were recorded using an Agilent GCMSD 7820*A*/5977B system.

### Notes

H_2_O_2_ and TBHP are strong oxidants and can undergo rapid exothermic decomposition, particularly in the presence of transition-metal catalysts. All reactions were conducted behind blast shields in a fume hood with external temperature monitoring. Vials used PTFE-lined caps to prevent the pressure buildup. Postreaction mixtures containing residual peroxides were quenched with 0.1 M Na_2_S_2_O_3_ solution prior to GC analysis or disposal. No thermal runaway, explosions, or fires occurred over >20 reaction repetitions under these controlled conditions.

### General Synthetic Procedure for **1–3**

The compounds were obtained according to the following synthetic procedure: 1 mmol of respective hydrazide and 1 mmol of respective salicylaldehyde were heated at 60 °C in 12 mL of methanol for 10 min, resulting in a yellow solution. Then 1 mmol of Cu­(NO_3_)_2_·3H_2_O was added, and the mixture was heated for an additional 15 min. The change of color to green was observed. After a few days, the crystals suitable for X-ray structure measurement were taken directly from the solution. The precipitate formed was filtered off, washed with methanol, and dried in air.

1. [{Cu­(L1)­(MeOH}­{Cu­(L1)­(MeOH)}]­(NO_3_)_2_, L1 = Schiff base ligand based on indole 3-acetic hydrazide and 5-chloro-salicylaldehyde; yield: 76%, elemental analysis calculated for **1**·H_2_O: C, 43.91; H, 3.48; N, 11.38%, found: C, 44.05; H, 3.56; N, 11.40%; magnetic moment 0.96 μ_B_. IR-ATR (cm^–1^): 3395 ν­(O–H), 2950 ν­(NH), 1602 ν­(C=O), 1569 ν­(C=N), 1377 ν­(NO_3_). UV–vis reflectance spectrum: (BaSO_4_) 684, 388, 286 nm, UV–vis spectra in solutions: (MeCN): 404 nm, (acetone): 402 nm, (DMSO): 391 nm, (MeOH): 389 nm; ε_389_ = 1.54·10^4^ dm^3^·mol^–1^·cm^–1^, (EtOH): 263, 386 nm.

2. [Cu­(L2)­(MeOH)_2_]­NO_3_, L2 = Schiff base ligand based on benzhydrazide and 3-bromo-5-chloro-salicylaldehyde; yield: 81%, elemental analysis calculated: C, 35.42; H, 3.16; N, 7.75%, found: C, 35.43; H, 2.71; N, 7.70%; magnetic moment 1.72 μ_B_. IR-ATR (cm^–1^): 3175 ν­(O–H), 2969 ν­(NH), 1625 ν­(C=O), 1548 ν­(C=N), 1384 ν­(NO_3_). UV–vis reflectance spectrum: (BaSO_4_) 714, 423, 305 nm, UV–vis spectra in solutions: (MeCN): 413, 290, 261 nm, (acetone): 409, 339 nm, (DMSO): 399, 340, 326, 308 nm, (MeOH): 411, 314, 304 nm; ε_411_ = 1.24·10^4^ dm^3^·mol^–1^·cm^–1^ (EtOH): 396, 334, 320, 261 nm.

3. [Cu­(L3)­(NO_3_)­(MeOH)], L3 = Schiff base ligand based on p-toluic hydrazide and 3-bromo-5-chloro-salicylaldehyde; yield: 75%, elemental analysis calculated: C, 35.51; H, 2.98; N, 7.76%, found: C, 35.40; H, 2.67; N, 7.66%; magnetic moment 1.62 μ_B_. IR-ATR (cm^–1^): 3453 ν­(O–H), 2949 ν­(NH), 1618 ν­(C=O), 1546 ν­(C=N), 1504, 1294, 1039 ν­(NO_3_). UV–vis reflectance spectrum: (BaSO_4_) 701, 417, 264 nm, UV–vis spectra in solutions: (MeCN): 412, 337, 317, 292, 266 nm, (acetone): 408, 394 nm, (DMSO): 414, 400, 340, 328, 311 nm, (MeOH): 412 nm; ε_412_ = 1.18·10^4^ dm^3^·mol^–1^·cm^–1^ (EtOH): 412, 318, 306, 266 nm.

### Structural Studies

Diffraction intensity data for the single crystals of the three new compounds were collected at 100(2) K on a Rigaku XtaLAB Synergy-S diffractometer with mirror-monochromated Mo Kα radiation (λ = 0.71073 Å) for **2** and Cu Kα radiation (λ = 1.54184 Å) for **1** and **3**. All data are presented in Table S1. Cell refinement and data reduction were performed using firmware.^21^ The positions of all non-hydrogen atoms were determined using SHELXT software.^22^ All non-hydrogen atoms were anisotropically refined using a weighted full least-squares matrix on *F* ^2^.^23^ All H atoms attached to C atoms were placed in idealized geometries and refined using a riding model with Uiso­(H) fixed at 1.2 Ueq (*C*_arom_) and 1.5 Ueq (*C*_methyl_). The drawings were made with Diamond version. 5.0.0.^24^ Crystal structure data and refinement are presented in Table S1.

Powder X-ray diffraction (PXRD) patterns of the dried powders were recorded on a Malvern PANalytical Aeris diffractometer equipped with a copper lamp (λ_CuKα_ = 1.5406 Å) at 40 kV and 15 mA and a Ni-filter. Standard measurements were done for 2θ = 5°–90° with a 2θ step of 0.02° and a counting time of 23 s.

### Scanning Electron Microscopy (SEM)

The microstructure of the powders was tested using scanning electron microscopy (SEM), and energy dispersive X-ray spectroscopy (EDS) analysis was performed with a ThermoFisher SCIOS 2 LoVac electron microscope.

### Alkane Oxidation

Peroxidative oxidation reactions were carried out in 20 mL Pyrex vials equipped with magnetic stirring and immersed in a thermostated oil bath at atmospheric pressure. In a typical procedure, a mixture of acetonitrile (1.5 mL), alkane substrate (toluene, 100 μmol), copper­(II) catalyst (1 μmol), and oxidant (either 30% aqueous H_2_O_2_ or 5.5 M *tert*-butyl hydroperoxide (TBHP) in dodecane, 10 mmol) was stirred at 80 °C for 1–24 h. After the reaction time, chlorobenzene (10 μL) was added as an internal standard, and the mixture was filtered through a short silica plug. Conversion and product analysis were determined by gas chromatography using mesitylene as an additional internal standard.

### Alkene Oxidation

Peroxidative oxidation reactions of alkenes were performed in 20 mL Pyrex vials equipped with magnetic stirring and immersed in a thermostated oil bath at atmospheric pressure. In a typical experiment, acetonitrile (1.5 mL), alkene substrate (styrene, 100 μmol), copper­(II) catalyst (1 μmol), and oxidant (30% aqueous H_2_O_2_ or 5.5 M *tert*-butyl hydroperoxide in dodecane, 10 mmol) were combined and stirred at 80 °C for 1–24 h. After completion, chlorobenzene (10 μL) was added as an internal standard, and the reaction mixtures were filtered through a short silica plug. Substrate conversion and product distribution were analyzed by gas chromatography using mesitylene as an additional internal standard.

### Hot Filtration Test (Toluene Oxidation)

A hot filtration test was performed to confirm the homogeneous nature of the catalysis in toluene oxidation using complex **1** (1 μmol). After 3 h at 80 °C, the reaction mixture was filtered through a short silica plug (80 °C, N_2_ atmosphere). The filtrate (1.5 mL CH_3_CN) was reheated to 80 °C, charged with additional H_2_O_2_ (10 mmol), and stirred for 21 h. Conversion and product analysis were determined by gas chromatography using mesitylene as an additional internal standard.

### Hot Filtration Test (Styrene Oxidation)

A hot filtration test was performed to confirm the homogeneous nature of the catalysis in styrene oxidation using complex **1** (1 μmol). After 3 h at 80 °C, the reaction mixture was filtered through a short silica plug (80 °C, N_2_ atmosphere). The filtrate (1.5 mL CH_3_CN) was reheated to 80 °C, charged with additional H_2_O_2_ (10 mmol), and stirred for 21 h. Conversion and product analysis were determined by gas chromatography using mesitylene as an additional internal standard.

### Bacterial Strains

Synthesized complexes were evaluated for their in vitro tuberculostatic potency against the reference strain *M. tuberculosis* H37Rv ATCC 25618, which is susceptible to all TB drugs, as well as two clinical *M. tuberculosis* strains isolated from tuberculosis patients. One of the clinical strains was MDR-TB, resistant to isoniazid and rifampicin (*M. tuberculosis* 210/21), while the other was resistant to isoniazid (3715).

### In Vitro Antitubercular Activity

The minimum inhibitory concentration (MIC) values were determined using the broth microdilution method according to CLSI M24, third ed.^25^ Investigations were performed in 96-well microtiter plates by the 2-fold serial microdilution using Middlebrook 7H9 Broth medium (Beckton Dickinson). The medium was supplemented with Tween 80 (Sigma-Aldrich) and oleic acid-albumin-dextrose-catalase (OADC) supplement (Becton Dickinson) according to the manufacturer’s instructions. The inoculum was prepared from fresh LJ culture in Middlebrook 7H9 Broth medium with OADC and Tween 80, adjusted to 0.5 McFarland standard with a nephelometer (Becton-Dickinson), and diluted 1:100. The stock solution of the tested agent was prepared in DMSO. The stock solutions 200,000 μg/mL of a tested agent prepared in dimethyl sulfoxide (DMSO) and diluted in sterile water to a concentration of 20,000 μg/mL were used to prepare series of different compound concentrations using the Middlebrook 7H9 broth medium. Subsequently, 100 μL of microbial suspension was added to each well. The final inoculum of all studied microorganisms was approximately 5 × 10^5^ CFU/mL, and concentrations of tested agents ranged from 5000 to 2.44 μg/mL. The highest concentration of DMSO used in the samples was tested to evaluate any possible bactericidal effect of the solvent.

A growth control containing no antibiotic and a sterile control without inoculation were also prepared on each plate. Four replicates were performed for each concentration of complexes and the control. The plates were incubated at 37 °C for 21 days. After the incubation period, 30 μL of Alamar blue solution was added to each well, and the plate was reincubated for 24 h. Growth is indicated by the color change from blue to pink, and the lowest concentration of compound that prevented the color change was noted as its MIC.^26,27^ Isoniazid (INH), rifampicin (RIF), and ethambutol (EMB) as reference drugs were used for comparison. Concentrations of drugs ranged from 512 to 0.0625 μg/mL.

### Checkerboard Assay

Synergistic interactions between synthesized complexes and antibiotics, INH, RIF, and EMB, were examined by the checkerboard test.^28^ For the checkerboard test, the MIC of each antibiotic was determined alone and in combinations against each isolate in one 96-well plate. Positive growth controls were performed in wells without an antibiotic to check for the existence of turbidity. The fractional inhibitory concentration index (FICI) was calculated for each combination of two agent concentrations according to the following formula:

$$\mathrm{FICI}=\left({\mathrm{MIC}}_{A/B}/{\mathrm{MIC}}_{A}\right)+\left({\mathrm{MIC}}_{B/A}/{\mathrm{MIC}}_{B}\right)$$

where MIC_A_ = MIC of the compound A alone, MIC_A/B_ is the MIC of compound A in combination with compound B, and MIC_B_, MIC_B/A_ is defined analogously as compound A.

Total synergism (FICI ≤ 0.5), additivity (0.5 < FICI ≤ 1), no effect (1 < FICI ≤ 4) or antagonism (FICI ≥ 4) between two compounds were deduced from the values of the FICI.^29^

### Theoretical Calculations

DFT calculations were carried out for the [{Cu­(L1)­(MeOH}­{Cu­(L1)­(MeOH)}]·(NO_3_)_2_ (**1**), [Cu­(L2)­(MeOH)_2_]·NO_3_ (**2**), and [Cu­(L3)­(NO_3_)­(MeOH)] (**3**), complexes. The main part of the calculations has been performed based on the crystal structure. However, geometry optimization has been performed as well. All calculations have been done in the Amsterdam Density Functional (ADF) program, version 2019.^30^ For each structure, B3LYP as an exchange-correlation functional^31,32^ has been applied. The Grimme dispersion correction has been used.^33^ Solvation effects were modeled with the discrete Conductor-like Screening Model (COSMO).^34^ The zero-order regular approximation (ZORA) to the Dirac equation was used.^35^ For all the atom types in the system, the triple-ζ basis set (TZP) was used. To visualize results of geometry optimization, for example, electronic features of considered systems like molecular electrostatic potential (MEP)^36^ or molecular orbitals (MOs), the ADF View package was used. Bonding between ligands and the metal center was analyzed with the Ziegler-Rauk bond energy decomposition scheme (ETS method).^37,38^ In this approach, the overall bonding energy is decomposed as Δ*E*_bonding_ = Δ*E*_orb_ + Δ*E*_steri*c*_ + Δ*E*_disp_ = Δ*E*_orb_ + (Δ*E*_elstat_ + Δ*E*_Pauli_) + Δ*E*_disp_; where Δ*E*_bonding_ is the total interaction energy of fragments (in the geometry of the complete interacting system). The orbital interaction term, Δ*E*_orb_, describes the energetic effect related to the charge transfer between the fragments, and their internal polarization (thus, leading to the formation of the optimized orbitals of the whole system), and the Δ*E*_disp_ is the dispersion correction based on the Grimme approximation. Δ*E*_steric_ can be decomposed into the electrostatic term, Δ*E*_elstat_, describing the electrostatic interaction between the unperturbed fragments and the Pauli repulsion term, Δ*E*_Pauli_ (mutual orthogonalization of the fragment orbitals; without any charge transfer between the fragments).

## Results and Discussion

### General Remarks to **1–3**

Three copper­(II) complexes with tridentate ONO-donating Schiff base ligands were synthesized via one-pot reaction (Scheme 1). Single-crystal X-ray diffraction reveals distinct coordination spheres for each complex. Complex **1** is an ionic dimer featuring coordination of a methanol molecule and the tridentate ligand to copper­(II), with the nitrate ion residing outside the coordination sphere. Complexes **2** and **3** are monomeric and differ in nitrate binding; nitrate serves as a counterion in **2** but as a ligand in **3**, replacing one coordinated methanol molecule. The compositions of the compounds were also confirmed by elemental analyses, which are in agreement with the results of diffraction methods. The magnetic susceptibilities of the complexes are close to the theoretical values for copper­(II) d^9^; for compound **1,** the value is given per one metal center.

**Scheme 1:**
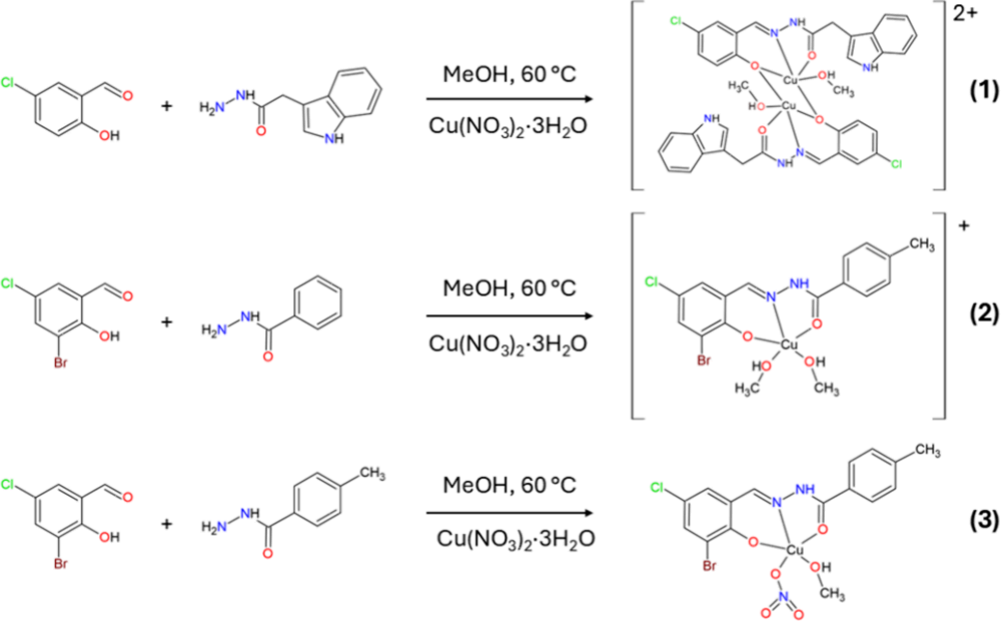
Scheme of Syntheses for Complexes **1**–**3** (Up to Down). In **1** and **2** the NO_3_^–^ Counterions were Omitted for Clarity

### Spectral Characterization

Infrared (IR) and UV–Vis spectra are provided in the Supporting Information. The IR spectra (Figures S1–S3) are consistent with the crystal structure and elemental analysis. Characteristic absorptions arise from methanol (broad features above 3000 cm^–1^) and nitrate anions. IR spectra of complexes **1** and **2** reveal intense bands at 1376 and 1384 cm^–1^, respectively. This is consistent with literature data for complexes in which nitrate acts as a counterion.^39–41^ In complex **3**, nitrate acts as a ligand, and three bands are observed at 1504, 1294, and 1039 cm^–1^, associated with the ν­(N=O), ν_asym_(NO_2_), and ν_sym_(NO_2_) vibrations, which have also been previously observed for copper­(II) complexes of a similar type.^39^ The remaining spectral features are dominated by absorptions of the organic ligand, including C=O and N–H modes at approximately 1600 and 3100 cm^–1^, respectively.

The UV–Vis spectra of the complexes were recorded in acetone, DMSO, acetonitrile, methanol, and ethanol (Figures S4 and S6). All compounds exhibit good solubility in these solvents and display an intense band near 400 nm, attributed to metal-to-ligand charge transfer (MLCT). The exact position of this band shows a small solvent dependence. Additional features below 350 nm can be assigned to ligand-centered transitions. The stability of complexes **1**–**3** was studied during 105 min of serial absorbance measurements, and the spectra presented in Figures S7 and S8 indicate the stability of the compounds. Additionally, measurements were performed 24 and 48 h after the solutions were prepared, and no changes in the spectra were observed. Solid-state diffuse reflectance spectra were also measured using BaSO_4_ as a reference (Figure S9). The MLCT band at ∼400 nm remains clearly visible, accompanied by a broad feature at ∼700 nm that can be attributed to *d–d* transitions, which are masked in solution by the high molar absorptivity of the MLCT band.

### CV Study

Cyclic voltammetry measurements were performed using a reference electrode for nonpolar solvent – Ag/AgCl, 0.1 M AgNO_3_ in MeCN. DMSO was used as the solvent, and 0.1 M [Bu_4_N]­(PF_6_) solution as the electrolyte. Measurements were performed in the range of −1.9 to 0.5 V using several scan rates: 400, 200, 100, and 50 mV/s. The results are shown in Figure 1.

**Figure 1:**
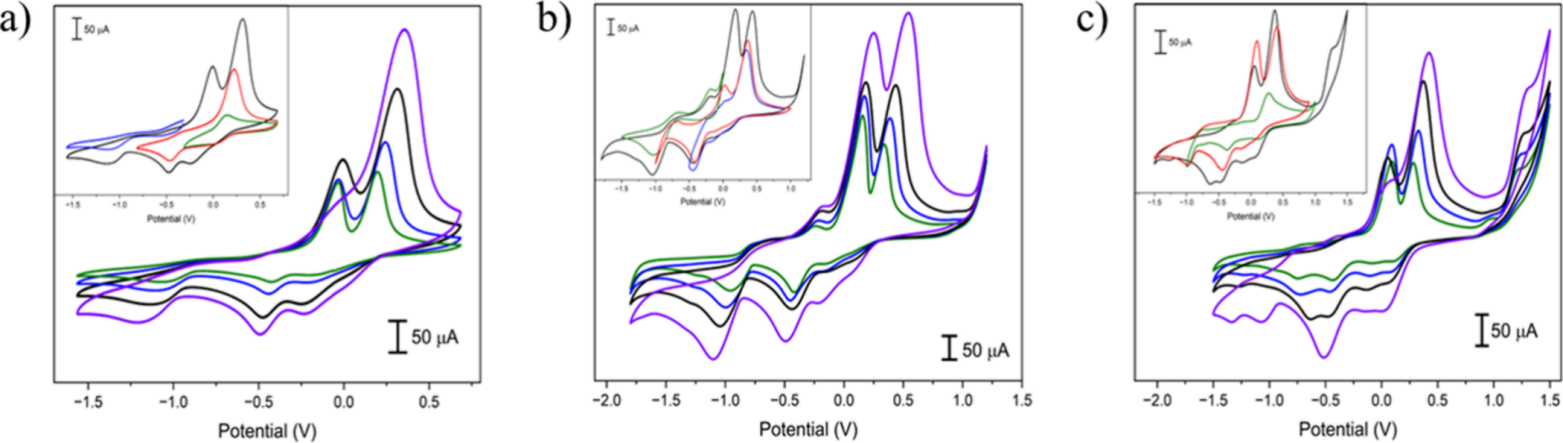
Cyclic voltammograms of complex (a) **1** (b) **2** (c) **3** in full range for different scan rate [mV/s]: 50 (green line), 100 (blue line), 200 (black line), 400 (violet line); and voltammograms in different scan range for 200 mV/s.

The obtained voltammograms had peaks located at the same potential values. However, they differ slightly in intensity and shape, which may be due to the different structures of the compounds studied. The reduction peaks located at approximately −0.45 and −0.15 V correspond to the reduction of copper­(II) to copper­(I) and copper­(I) to copper(0), respectively. These are reversible processes with oxidation peaks located at approximately 0.10 and 0.30 V.^42,43^ For compounds **1** and **3**, the first of these oxidation peaks has an intensity lower than that of the second peak and, additionally, disappears at high scanning speeds (400 mV/s). However, the corresponding peaks for compound **2** have comparable intensities and do not disappear with an increase in speed. This may be related to the monomeric and ionic structures of compound **2**, which allow the described copper redox processes to occur more easily than for the dimeric compound **1** or compound **3**, which has a nonionic structure.

The reversible peak at lower potential values (*E*_red_ = −1.00 and *E*_ox_ = −0.76 V) is associated with redox processes of the azomethyl group of the Schiff base ligands. An oxidation peak at approximately −0.25 V is also associated with this process.^44–46^ Described peaks are best formed for compound **2**, which may also be due to differences in the structures of the studied complexes.

### Crystal Structures

Compounds **1** and **2** crystallize in the triclinic system (P $\overset{-}{1}$ space group), while compound **3** is in monoclinic C 2/c. The asymmetric unit of **1** consists of a copper­(II) complex cation with an ONO-donor Schiff base (L1) and a methanol molecule as ligands (Figure 2a). A nitrate ion is located outside the coordination sphere. Figure 2b shows the dimeric structure of the compound, which contains Cu(1)–O(1) and Cu(1)–O(1)#1 bonds with a length of 1.958 and 1.972 Å, respectively (#1: −x + 2, −y + 1, −z + 1). In complex **2**, the additional methanol ligand is coordinated to the copper­(II) metal center, and it fills the coordination sphere with a coordination number 5 (Figure 3). The complex **2** is an ionic monomer, in contrast to complex **1,** which is in a dimeric form. In complex **3**, the tridentate Schiff base, methanol, and nitrate are coordinated to Cu­(II), which indicates that a neutral monomer has been obtained (Figure 4). In **1**–**3,** the central copper atom adopts a tetragonal pyramidal geometry. Slight deviations from the typical angle values for this type of system are due to the large size of the organic ligand and the simultaneous presence of a much smaller ligand, methanol. Selected bond lengths and angles are provided in Table S2 in the ESI. For the N(1)–C(7), the bond length is ca. 1.29 Å, which corresponds to a nitrogen–carbon double bond and confirms the presence of an imine bond in the organic ligand. Similarly, the O(2)–C(8) bond length in all compounds is about 1.26 Å, which indicates a double bond and, as a result, a mononegative Schiff base ligand (from the deprotonated hydroxyl group of the O(1) atom). Figure 5, shows the layered packing of compound **1** formed by hydrogen bonds (Table S3). The dimeric copper­(II) complex molecule connects with as many as six nitrate anions, while the nitrate ion binds with three cation molecules in copper­(II) complexes (Figure 6). In compounds **2** and **3,** the nitrate ions are primarily involved in hydrogen interactions that stabilize the structure, which is clearly visible in Figures 3 and 4 as well as in the ESI (Tables S4 and S5). Crystal packing for **2** and **3** is also supported in the Supporting Information file (Figures S10 and S11). For **1**–**3** (Figures S12–S14, ESI), the experimental PXRD patterns of bulk samples matched well with the simulated patterns based on single-crystal data, confirming phase purity and structural consistency. This correspondence indicates that the crystalline phase present in the bulk samples is consistent with that of the single crystals, demonstrating retention of the molecular and packing arrangements upon scale-up.

**Figure 2:**
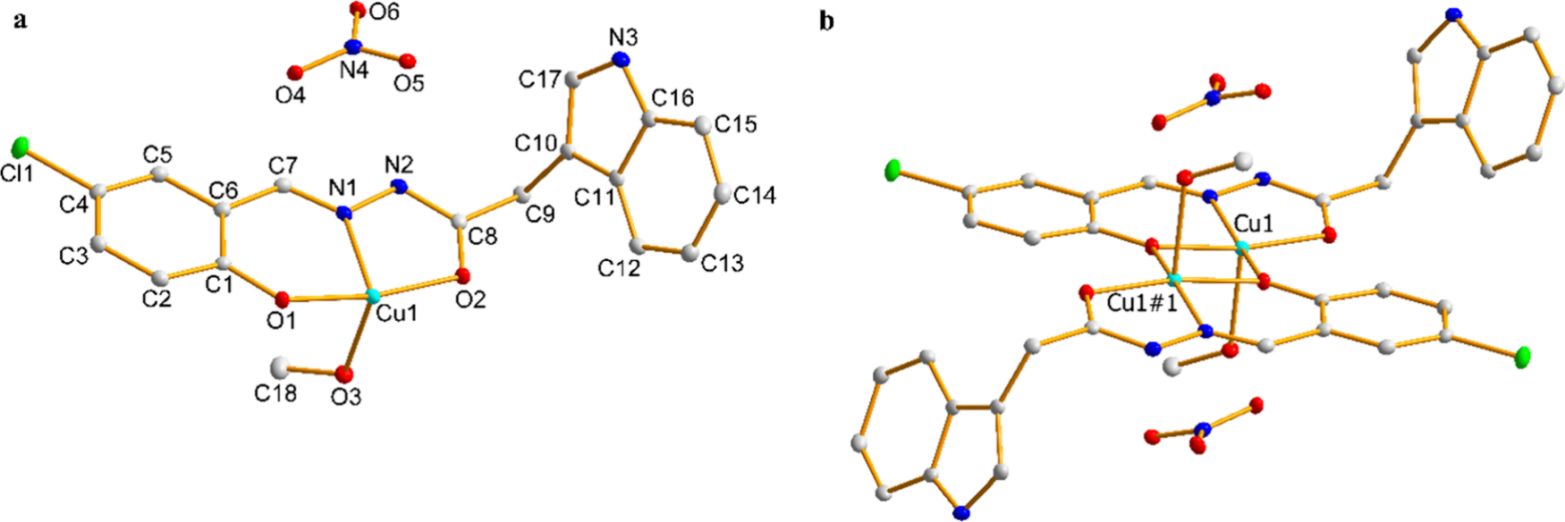
(a) Asymmetric part of the unit cell of **1**; (b) dimeric structure of compound **1**. The hydrogens were omitted for clarity. 30% elipsoid probability. Symmetry transformations used to generate equivalent atoms: #1 −x + 2, −y + 1, −z + 1.

**Figure 3:**
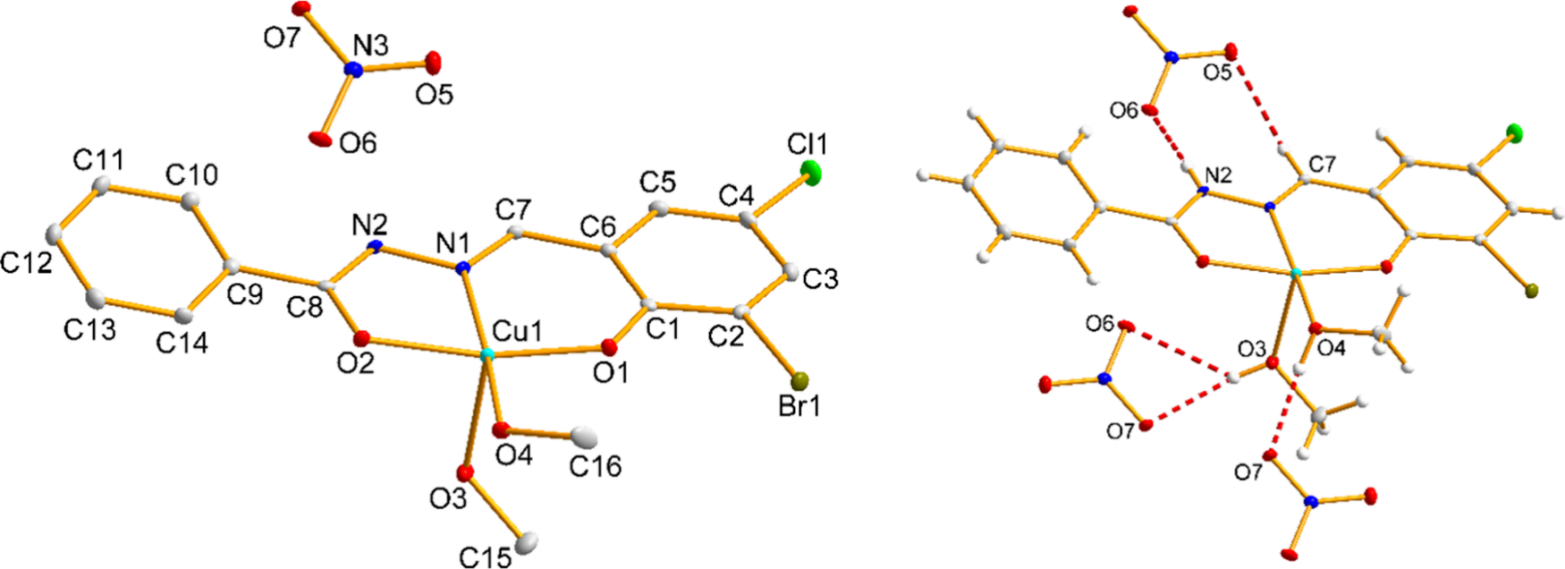
Asymmetric unit cell and hydrogen bonds in **2**. The hydrogens were omitted for clarity. 30% elipsoid probability.

**Figure 4:**
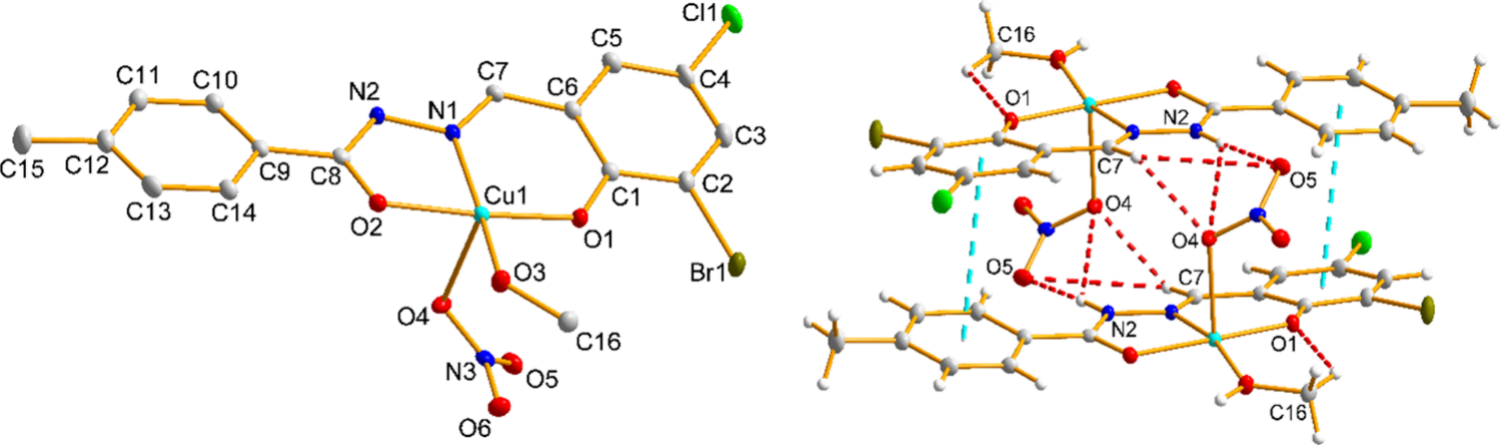
Asymmetric unit cell and hydrogen bonds in **3**. The hydrogens were omitted for clarity. 30% elipsoid probability.

**Figure 5:**
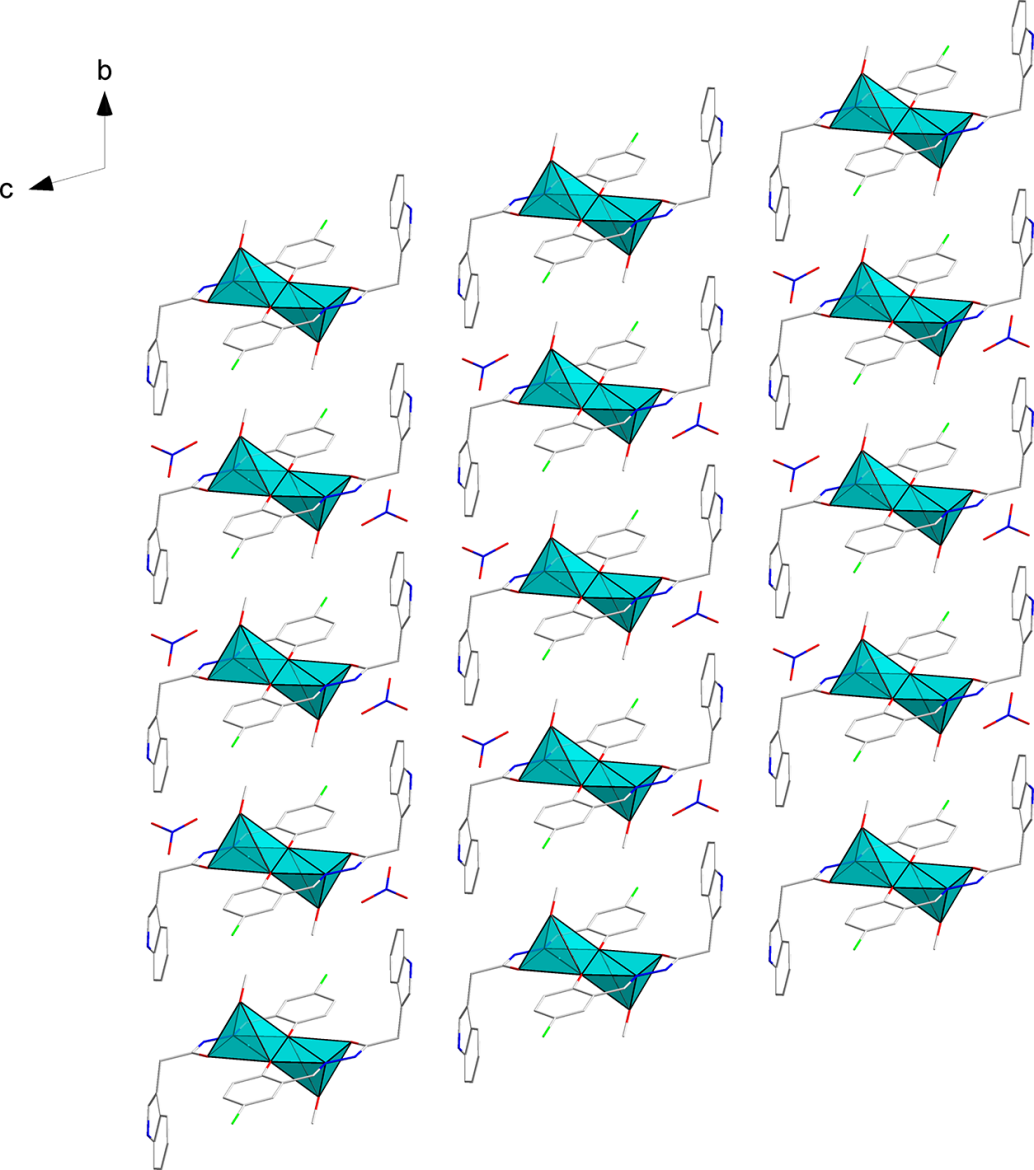
Layered packing of **1**.

**Figure 6:**
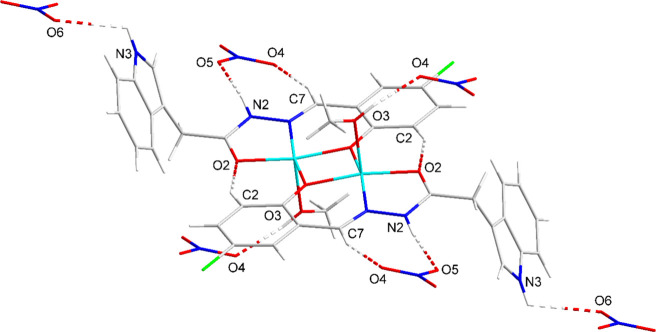
Hydrogen bonds in **1**.

The structural differences seen in compounds **1**–**3** can be explained by a balance between electronic and steric effects in the Schiff base structure and by the presence of other ligands bound to copper. Using 5-chloro- and 3-bromo-5-chloro-salicylaldehyde changes the electron density of the salicylidene/phenolate group through halogen effects and also affects the space around the donor atoms. In Cu­(II) Schiff base complexes, such changes can influence how the ligand coordinates, whether the complex forms one or two metal centers, and whether the phenolate oxygen atoms act as terminal or bridging donors. In this study, complex **1** forms a phenolate-bridged dimer, showing that the deprotonated phenolate can support a Cu–O–Cu bridge. In contrast, complexes **2** and **3** form monomers because methanol or nitrate ligands coordinate to copper, preventing bridge formation. Thus, the difference between dimeric and monomeric forms results from the combined effects of the substituents’ electronic and steric properties rather than a single factor. This agrees with earlier reports that even small structural changes in similar Cu­(II) systems can shift the balance between single- and double-metal complexes depending on ligand design, substituent pattern, and available coordination sites.

These structural observations are consistent with the electronic features obtained from DFT calculations, which confirm the dominant role of electrostatic stabilization in complex **1** and the progressive increase in the Pauli repulsion in complexes **2** and **3**. In particular, the DFT-derived charge distribution maps support the experimentally observed differences in nitrate coordination, revealing a more homogeneous electrostatic potential in **1** and more localized charge separation in **2** and **3**. Theoretical geometry relaxations also retained the experimentally determined coordination environments, further validating the rigidity of the ONO chelate and the influence of coordinated methanol or nitrate on the local molecular geometry.

### Scanning Electron Microscopy (SEM)

The SEM images of compounds **1**, **2**, and **3** (Figures S15, S17, S19) reveal distinct differences in morphology. Compound **1** exhibits well-formed, platelike crystals with smooth surfaces and sharp, regular edges, characteristic of highly crystalline materials. In contrast, compound **2** presents more blocky, irregular crystals with angular shapes, while compound **3** displays a fragmented, aggregate-like texture composed of smaller, platelike particles. The EDS spectra (Figures S16, S18, S20) confirm the presence of elemental components expected for each compound: for compound **1**, signals corresponding to Cl, C, N, O, and Cu are detected, while for compounds **2** and **3**, the spectra reveal distinct peaks for Br, Cl, and Cu alongside C, N, and O, in agreement with the formulas established by X-ray crystallography.

### DFT Results

To get some insight into the geometry and electronic structure of the studied compound, we have performed theoretical calculations with the density functional theory (DFT) method. One of the main goals of the theoretical study was to analyze the charge distribution in the investigated complexes. Further, we will discuss the ligand binding energies and the nature of the interactions using the Ziegler-Rauk energy decomposition analysis (ETS method).

It is worth noting that two approaches to partitioning the system have been discussed. The first partition analyzed the interaction of the counterion (NO_3_^–^) with copper complexes, as shown in the top part of Table 1. For molecule **1**, the interaction energy is the lowest (−87.82 kcal/mol) compared to compounds **2** and **3**. This is reflected in both the electrostatic component, representing the interaction between ions, and the orbital interaction component, which is associated with the charge transfer characteristic for hydrogen bonding. In system **2**, both hydrogen bonding and electrostatic interactions are weakened due to the presence of a methanol molecule between the counterions and the metal center. The interaction between fragments in system **3** is the highest (the least stabilizing) due to the relatively high value of the Pauli repulsion component. This system is also the only one in which the counterion interacts directly with the metal center. Additionally, panel a) of Figure 7 depicts the charge transfer between the counterion and complex by presenting the differential electron density.

**Table 1:** Calculated Values of ETS-Energy Decomposition Analysis[Table-fn t1fn1] Performed on the [{Cu­(L1)­(MeOH}­{Cu­(L1)­(MeOH)}]·(NO_3_)_2_, [Cu­(L2)­(MeOH)_2_]·NO_3,_ and [Cu­(L3)­(NO_3_)­(MeOH)] (All Values in kcal/mol)

			**Δ*E***_**elstat**_	**Δ*E***_**pauli**_		
**fragment 1:**	**fragment 2:**	**Δ*E*_orb_**	**Δ*E***_**ster**_		**Δ*E*_disp_**	**Δ*E*_bond_**
{Cu(L1)(MeOH}{Cu(L1)(MeOH)} (NO_3_)	NO_3_	–12.88	–87.95	17.32	–4.31	–87.82
			–70.62			
[Cu(L2)(MeOH)_2_]	NO_3_	–5.92	–76.33	12.69	–3.97	–73.53
			–63.63			
[Cu(L3) (MeOH)]	NO_3_	–11.39	–82.95	26.97	–5.34	–72.71
			–55.98			
{Cu(L1)}{Cu(L1)} (NO_3_)	(MeOH)_2_(NO_3_)	–37.28	–145.68	86.83	–19.20	–115.34
			–58.85			
[Cu(L2)]·	(MeOH)_2_(NO_3_)	–48.59	–142.72	93.47	–10.60	–108.44
			–49.25			
[Cu(L3)]	(MeOH)(NO_3_)	–46.21	–142.68	86.62	–8.93	–111.20
			–56.06			
[Cu](MeOH)_2_(NO_3_)	(L2)	–154.25	–270.14	234.30	–9.15	–253.17
			–35.84			
[Cu] (MeOH)(NO_3_)	(L3)	–148.59	–286.28	233.23	–8.61	–262.42
			–53.05			

a Δ*E*_bonding_ = Δ*E*_orb_ + Δ*E*_steric_ + Δ*E*_disp_ = Δ*E*_orb_ + (Δ*E*_elstat_ + Δ*E*_Pauli_) + Δ*E*_disp_.

**Figure 7:**
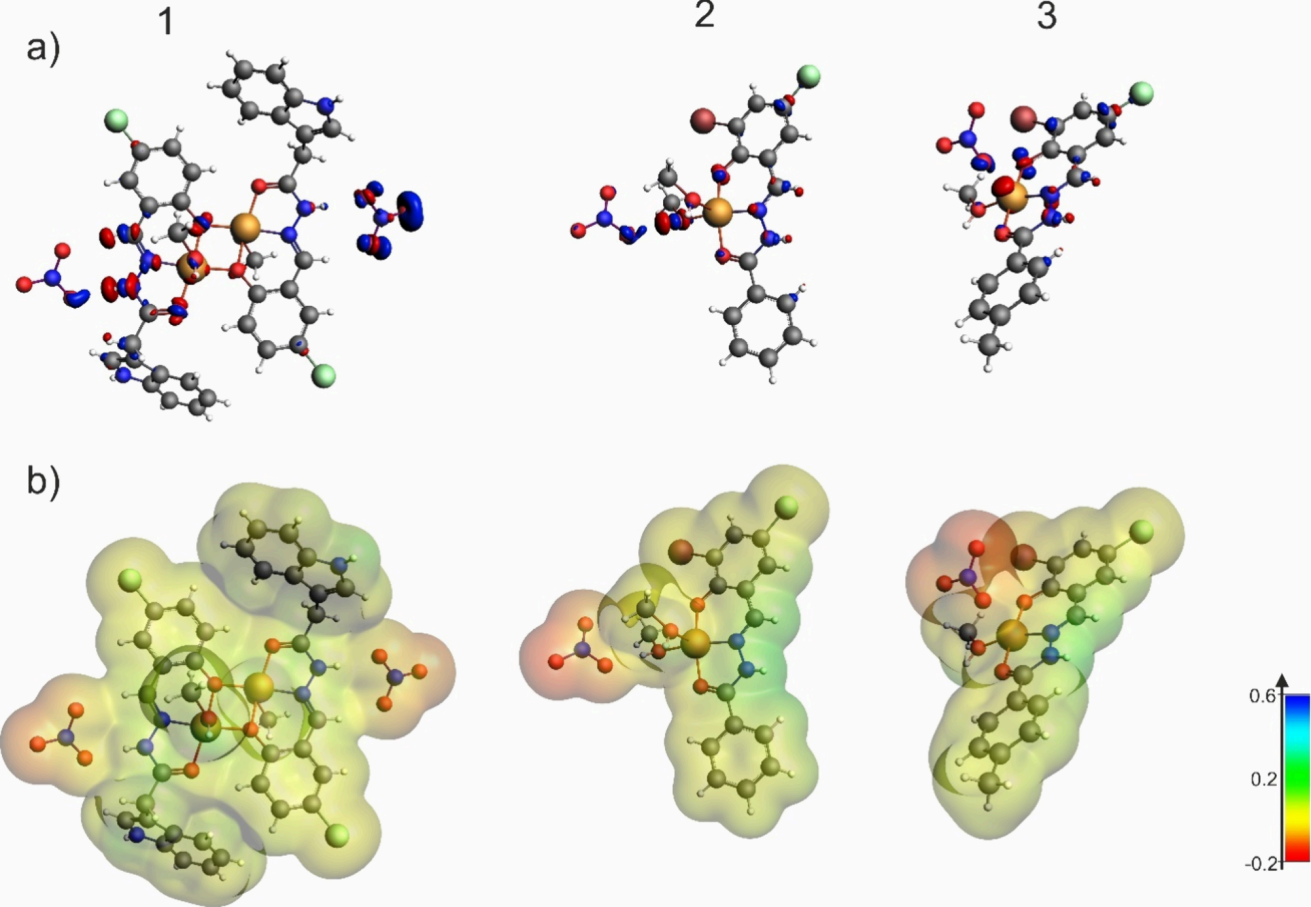
Panel (a) depicts deformation electron densities for studied systems (**1**, **2,** and **3**) based on the NO_3_^–^ fragment and the rest of the complexes; the contour values are 0.003 au.; blue represents density accumulation, and red represents density depletion. Panel (b) color representation of Molecular Electrostatic Potential (MEP) on electron density contours (Δρ = 0.002) for the studied complexes.

The second partition reflects the interaction between the counterion and the methanol molecules that interact with the copper center and chelating ligand. The results are summarized at the bottom of Table 1. As in the previous analysis, molecule **1** exhibited the most stabilizing interaction energy, which is attributed to the strong electrostatic nature of this interaction. For systems **2** and **3**, the stabilizing role of orbital interaction components increases, indicating significant charge transfer between fragments. This effect is compensated by increasing the Pauli repulsion component for both the **2** and **3** compounds.

To elucidate the difference between compounds **2** and **3,** we have studied the interaction between the copper fragment coordinated by solvent and nitrate ligands and the corresponding organic ligands L2 and L3. In both cases, the bonding is characterized by a very strong stabilization, as reflected by highly negative total bonding energies (−253.17 kcal mol^–1^ for L2 and −262.42 kcal mol^–1^ for L3). The interaction is mainly driven by pronounced electrostatic stabilization, with values of −270.14 kcal mol^–1^ for L2 and −286.28 kcal mol^–1^ for L3, which reflects strong charge–charge interactions between the copper center and the ligands. In addition, the orbital interaction terms are strongly stabilizing in both cases, emphasizing the significant covalent character of the Cu–ligand bonds, with slightly stronger orbital stabilization observed for L2. For both systems, orbital interaction terms are partially counterbalanced by large Pauli repulsion terms.

Dispersion contributions are comparatively small and play only a secondary role in the overall bonding. Notably, the [Cu­(MeOH)­(NO_3_)]–L3 interaction exhibits the most favorable total bonding energy among all analyzed fragment pairs, suggesting a particularly effective balance between electrostatic and orbital interactions for ligand L3. This indicates that the nature of the ligand has a decisive influence on the strength and character of metal–ligand bonding in the studied copper complexes.

The charge distribution depicted in panel (b) of Figure 7 by molecular electrostatic potential reflects quite a similar charge distribution for compounds **2** and **3**. The counterions accumulate a negative charge. The positive charge is mainly located around the center part of the chelating ligand. On the other hand, molecule **1** presents a more homogeneous charge distribution. However, it should be noted that formally for molecule **1**, the two counterions are present in the structure located on the opposite site. The detailed analysis of the frontier molecular orbitals, as well as reactivity descriptors, has been presented in the SI.

Although the present calculations were not designed to quantify substituent constants or bridge formation energies directly, they do support the view that complex **1** has a more favorable electrostatic organization for the observed dimeric arrangement, whereas **2** and **3** display more localized charge separation, consistent with monomer stabilization.

### Catalytic Activities

The catalytic behavior described below can be rationalized in part by the DFT-based energy decomposition analysis, which indicates that complex **1** possesses the most stabilizing ligand–metal interactions, dominated by electrostatic and orbital contributions. This enhanced stabilization correlates with the experimentally observed catalytic performance of **1**, as weaker interactions and increased Pauli repulsion in **2** and **3** may hinder efficient peroxide activation at the copper center. Moreover, the more homogeneous molecular electrostatic potential surface of **1**, revealed by DFT, may facilitate the formation of reactive Cu–OOH intermediates.

### Toluene Oxidation

The catalytic performance of the Cu­(II) complex **1** was evaluated for the homogeneous oxidation of toluene using hydrogen peroxide (H_2_O_2_) or tert-butyl hydroperoxide (TBHP). The reactions were conducted in acetonitrile at 80 °C, under typical conditions (reaction time of 3, 6, and 24 h for H_2_O_2_ and 1 h for TBHP). The results of the catalytic studies are summarized in Table 2.

**Table 2:** Oxidation of Toluene Using **1** ^a^

entry	time [h]	conversion [%][Table-fn t2fn2]	benz-aldehyde (%)	*o*-cresol (%)	phenylmethanol (%)	2-methylcyclohexa-2,5-diene-1,4-dione (%)	p-cresol (%)	TON^c^	TOF [h^-1^]^d^
1	3	31	10	9	1.5	6	5	31	10
2	6	30	11	10	1	1	7	30	5
3	24	34	13	12	1	1	7	34	1.5
4^e^	1	0						0	0

a Reaction conditions: toluene (100 μmol), **1** (1 μmol, 1%), CH_3_CN (1.5 mL), H_2_O_2_ (10 mmol), 80 °C.

bb Conversion was determined using GC analysis of the reaction mixture.

c Turnover number = (moles of benzaldehyde + *o*-cresol + phenylmethanol···)/mol of Cu catalys.

d Average turnover frequency in [mol [toluene­[mol^–1^ [Cu] h^–1^],

e Reaction conditions: toluene (100 μmol), **1** (1 μmol, 1%), CH_3_CN (1.5 mL), TBHP (10 mmol), 80 °C.

Complex **1** exhibited moderate catalytic activity in the oxidation of toluene with H_2_O_2_, with conversion levels ranging from 30% to 34%, depending on the reaction time (Scheme 2). After 3 h, the system achieved a 31% conversion, with benzaldehyde (10%) and o-cresol (9%) as the primary products. Minor products included p-cresol (5%), 2-methylcyclohexa-2,5-diene-1,4-dione (6%), and phenylmethanol (1.5%). The turnover number (TON) was calculated as 31, with a turnover frequency (TOF) of 10 h^–1^.

**Scheme 2:**
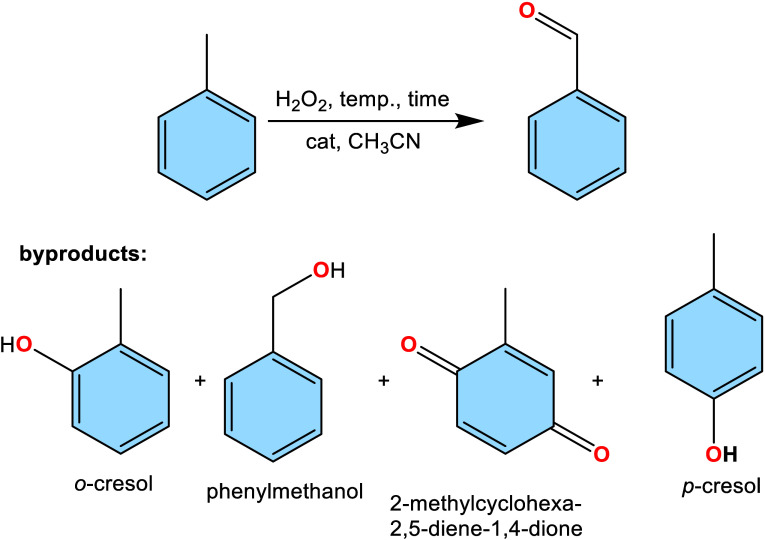
Oxidation of Toluene Using **1** ^a^

a Reaction conditions: toluene (100 μmol), 1 (1 μmol, 1%), CH_3_CN (1.5 mL), H_2_O_2_ (10 mmol), 80 °C

Benzaldehyde dominates (10–13%) over benzoic acid (absent or <0.1%), indicating efficient benzylic C–H abstraction to benzyl alcohol (detected at ∼1–1.5% level), followed by selective single oxidation rather than overoxidation to carboxylic acid. This limited further oxidation aligns with mild conditions (80 °C, excess H_2_O_2_) where Cu­(II)–OOH intermediates favor alcohol-to-aldehyde conversion without promoting aldehyde hydration or Cannizzaro-type reactions. o-Cresol (9–12%) significantly outpaces p-cresol (5–7%), suggesting preferential attack at the ortho position relative to the directing methyl group.

The Cu­(II)–OOH species likely acts as the active electrophile, suggesting preferentially attacking methyl-activated ortho positions despite 1,2-sterics, while minor p-cresol arises from less selective homolytic HO- addition. 2-Methylcyclohexa-2,5-diene-1,4-dione (∼6%) represents a quinone pathway from the overoxidized o-cresol tautomer. TBHP inactivity (0% conversion) confirms the requirement for unhindered H_2_O_2_-derived Cu­(II)–OOH, as steric bulk impedes substrate approach to the active site.

After 6 h, the overall conversion slightly decreased to 30%, although benzaldehyde and o-cresol yields modestly increased to 11% and 10%, respectively. Other oxidation products were observed at similar levels as in the shorter reaction time, while the TON and TOF declined to 30 and 5 h^–1^, respectively, indicating a decrease in catalytic efficiency.

After 24 h, conversion improved to 34%, with benzaldehyde and o-cresol reaching 13 and 12%, respectively. With a turnover frequency of 1.5 h^–1^.

The progressive loss of catalytic activity for complex 1, evident from declining TOF values (10 h^–1^ at 3 to 1.5 h^–1^ at 24 h) and marginal conversion increase (31–34%), despite rising benzaldehyde (10–13%) and o-cresol (9–12%) yields, signals catalyst deactivation (Table 2). This stems from over-reduction of Cu­(II) to inactive Cu­(I) under excess H_2_O_2_ (10 mmol, 80 °C), supported by quasi-reversible CV reduction at E_red_ ≈ −0.45 V (Cu­(II)/Cu­(I)) and postreaction μ_eff_ = 0 μ_B_ per Cu as well as. Solid-state diffuse reflectance spectra revealed that the absorption bands characteristic of Cu­(II) species at 700 nm, attributable to *d–d* transitions, had disappeared (Figure S21). This indicates the absence of Cu­(II) in the postcatalytic sample.

The ionic dimeric structure exacerbates this reductive pathway, which is common in homogeneous Cu–peroxide systems. Postreaction ATR-IR spectra confirm preservation of the ligand framework; however, a reduction from Cu­(II) to Cu­(I) is evident, as indicated by μ_eff_ = 0 μ_B_. No ligand hydrolysis is observed. The contrast between H_2_O_2_ and TBHP (0% conversion, Table 2) underscores heterolytic O–O cleavage for the Cu­(II)–OOH species, which is impeded by the bulky t-BuOOH ligand.

The bulky tert-butyl group of TBHP (compared to the accessible hydroperoxo moiety of H_2_O_2_) sterically impedes substrate approach to the constrained dimeric active site of complex **1**. Postreaction ATR-IR spectra reveal a new weak band at 1178 cm^–1^ attributed to ν­(O–O) vibration (Figure S22), diagnostic of Cu-hydroperoxo formation specifically with H_2_O_2_ and absent in TBHP control experiments. This suggests that TBHP cannot generate the requisite [Cu­(II)­(L)­(OOH)] intermediate due to steric occlusion. The comparable aqueous redox potentials of both oxidants (H_2_O_2_/OH^–^ E° = 0.87 V; t-BuOOH/t-BuO^–^ E° = 1.60 V, though similar in MeCN) rule out differences in thermodynamic driving force. Instead, the observed selectivity arises from activation barrier disparities: H_2_O_2_ undergoes facile heterolytic O–O cleavage facilitated by Lewis-acidic Cu­(II), whereas TBHP’s electron-donating tert-butyl group weakens O–O bond activation and favors unproductive homolysis in the absence of substrate coordination.

Cu­(II) complex **1** exhibits catalytic activity comparable to other homogeneous systems in the literature, particularly in terms of selectivity toward benzaldehyde and o-cresol. Hot filtration and postreaction analyses confirm homogeneous catalysis and ligand stability. The lack of activity with TBHP distinguishes it from some reported systems and may reflect specific electronic or structural features of the complex. In the study by Pombeiro et al.^3^ Cu­(II) complexes structurally related to the one used in this work showed catalytic activity in the oxidation of toluene with H_2_O_2_, achieving total product yields of up to 11% after 3 h at 80 °C. The main products, benzaldehyde and o-cresol, were consistent with those observed in our study, indicating a similar oxidation pathway. Notably, in their system, TBHP also led to product formation (up to 6.6% total yield), unlike complex **1**, which showed no conversion under TBHP conditions.

### Styrene Oxidation

The catalytic activity of Cu­(II) complex **1** was further evaluated for the oxidation of styrene using hydrogen peroxide (H_2_O_2_) as the oxidant in acetonitrile at 80 °C (Scheme 3). Reactions were monitored over various time intervals (3, 6, and 24 h), and the corresponding conversions and product distributions were determined via GC analysis (Figure S23). A control experiment at 60 °C was also conducted for comparison. The results of the catalytic studies are summarized in Table 3.

**Scheme 3:**
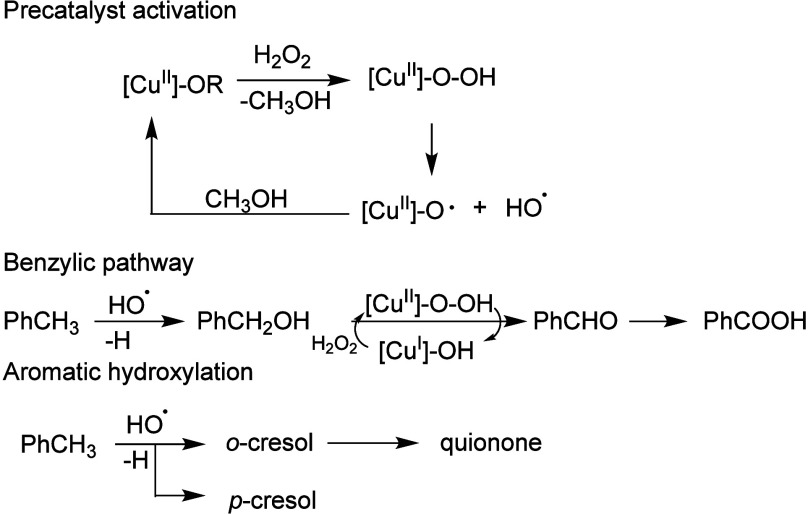
Proposed Reaction Pathway of Oxidation of Toluene Using **1**

**Table 3:** Oxidation of Toluene Using **1** ^a^

entry	oxidizer	Time [h]	Conversion [%]^b^	Benzaldehyde (%)	Styrene oxide (%)	Acetophenone (%)	Benzoic acid (%)	2,2-dihydroxy-1-phenylethan-1-one (%)	TON^c^	TOF [h^-1^]^d^
1	H_2_O_2_ ^e^	3	1%	1					1	0.33
2	H_2_O_2_	3	16	11	0.77	1.24	0.7	3.29	17	5.67
3	H_2_O_2_	6	17	8.8	1.5	1	1.5	3.5	17	2.83
4	H_2_O_2_	24	19	12	0	1.6	1.3	3.75	19	0.79

a Reaction conditions: styrene (100 μmol), **1** (1 μmol, 1%), CH_3_CN (1.5 mL), H_2_O_2_ (10 mmol), 80 °C.

b The conversion was determined using GC analysis of the reaction mixture.

c Turnover number = (moles of benzaldehyde + styrene oxide + acetophenone ···)/mol of Cu catalys.

d Average turnover frequency in [mol of toluene]­[mol^–1^ Cu]­[ h^–1^].

e Reaction conditions: styrene (100 μmol), **1** (1 μmol, 1%), CH_3_CN (1.5 mL), H_2_O_2_ (10 mmol), 60°C.

At 60 °C (Entry 1), the system exhibited minimal activity, yielding only 1% conversion with benzaldehyde as the sole detectable product, indicating that elevated temperatures are necessary to activate the catalyst. At 80 °C and 3 h (Entry 2), the conversion increased significantly to 16%, with benzaldehyde (11%) as the dominant product, accompanied by minor amounts of styrene oxide (0.77%), acetophenone (1.24%), benzoic acid (0.7%), and 2,2-dihydroxy-1-phenylethan-1-one (3.29%). The turnover number (TON) was calculated as 17, and the turnover frequency (TOF) was 5.67 h^–1^.

The progressive loss of catalytic activity for complex 1 in styrene oxidation, evident from declining TOF values (5.7 h^–1^ at 3 to 0.8 h^–1^ at 24 h) and marginal conversion increase (16–19%), despite rising benzaldehyde (8.8–12%) yields, signals catalyst deactivation. This stems primarily from over-reduction of Cu­(II) to inactive Cu­(I) under excess H_2_O_2_ (10 mmol, 80 °C), supported by quasi-reversible CV reduction at E_red_ ≈ −0.45 V (Cu­(II)/Cu­(I)), postreaction effective magnetic moment μ_eff_ = 0 μ_B_ per Cu, and disappearance of the Cu­(II) *d–d* transition band at 700 nm in solid-state diffuse reflectance spectra. The ionic dimeric structure exacerbates this reductive pathway, common in homogeneous Cu-peroxide systems, while postreaction ATR-IR confirms ligand framework preservation without hydrolysis.

Prolonging the reaction time to 6 h (entry 3) resulted in a slight increase in conversion (17%) but a reduction in benzaldehyde selectivity (8.8%), with a concomitant increase in byproduct formation, notably styrene oxide (1.5%) and dihydroxyketone (3.5%). At 24 h (entry 4), conversion reached 19%, with benzaldehyde yield rising to 12%. However, styrene oxide was no longer detected, and byproducts such as acetophenone, benzoic acid, and the dihydroxyketone persisted, suggesting the progressive overoxidation of initial products.

The observed trend in TOF, which declined from 5.67 h^–1^ (3 h) to 0.79 h^–1^ (24 h), indicates a gradual loss of catalytic efficiency over time, likely due to catalyst degradation or peroxide decomposition. Overall, complex **1** demonstrates moderate activity in styrene oxidation with the highest efficiency and selectivity observed at shorter reaction times. The disappearance of styrene oxide at prolonged times suggests its instability under the reaction conditions, emphasizing the need for careful optimization of the reaction parameters to enhance selectivity and suppress overoxidation.

Hot filtration tests confirm the homogeneous nature of catalysis with complex **1**. After 3 h reaction at 80 °C, the mixture was filtered through a short silica plug under N_2_ atmosphere (80 °C), and the filtrate (1.5 mL CH_3_CN) was reheated to 80 °C with fresh H_2_O_2_ (10 mmol) for 21 h (Scheme 4). No additional substrate conversion occurred, ruling out catalytically active Cu leaching. Postreaction analysis reveals catalyst deactivation via over-reduction of Cu­(II) to inactive Cu­(I), which was confirmed by the observation of μ_eff_ = 0 μ_B_ per Cu and disappearance of the 700 nm *d-d* band in solid-state diffuse reflectance spectra. ATR-IR confirms ligand integrity with preserved C=N (1569 cm^–1^) and C=O (1602 cm^–1^) bands, indicating no hydrolysis under reaction conditions.

**Scheme 4:**
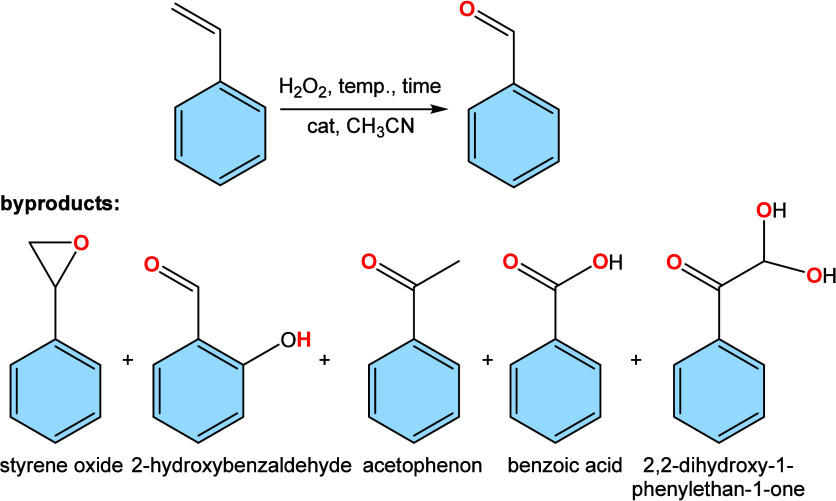
Oxidation of Styrene Using **1** ^a^

a Reaction conditions: styrene (100 μmol), **1** (1 μmol, 1%), CH_3_CN (1.5 mL), H_2_O_2_ (10 mmol), 80 °C

### Antimycobacterial Activity and Drug Interaction Studies

Because of the growing problem of multidrug resistance in mycobacteria, it is essential to evaluate new therapeutic combinations that include both established antitubercular drugs and newly synthesized compounds. Combination therapy is already a cornerstone of conventional tuberculosis (TB) treatment, offering improved outcomes compared to monotherapy. One of the primary benefits of drug combinations is the reduction in the minimum inhibitory concentrations (MICs) of conventional drugs, which can subsequently lead to fewer side effects.

In this context, we have assessed the antitubercular potential of newly synthesized compounds alone and in combination with one of three reference drugs. The aim was to determine possible interactions, including synergism, additive, or antagonistic effects.

To investigate the antimycobacterial properties of the synthesized complexes **1**–**3**, MIC values were determined. Complexes **1**–**3** inhibited the growth of the reference *M. tuberculosis* H_37_R_v_ strain at MICs ranging from 312.5 μg/mL. The same MIC values were observed for both the multidrug-resistant strain (210/21) and the isoniazid-monoresistant strain (3715) (Table 4). The MICs of complexes **1**–**3** were markedly higher compared to those of the standard drugs, indicating lower tuberculostatic potency under the tested conditions.

**Table 4:** Activity of Complexes **1-3** against *Mycobacterium tuberculosis* Strains; Minimal Inhibitory Concentrations (MIC, μg/mL)

Comp.	*M. tuberculosis* H_37_Rv ATCC 25618	*M. tuberculosis* 210/21 (multidrug-resistant)	*M. tuberculosis* 3715 (INH-monoresistant)
1	312.5	312.5	312.5
2	312.5	312.5	312.5
3	312.5	312.5	312.5
isoniazid (INH)	0.0625	16	1
rifampicin (RIF)	0.25	512	0.25
ethambutol (EMB)	0.5	4	0.5

The identical MIC values for complexes **1**–**3** do not allow a clear structure–activity relationship to be determined in this series. All three compounds contain the same Cu­(II)–ONO core and differ only in small changes in the outer parts of the ligands and in their solid-state nuclearity. Therefore, the antimycobacterial activity is most likely controlled mainly by the common copper coordination motif rather than by these minor structural differences.

In Cu­(II) Schiff base complexes, biological activity usually results from several factors acting together, such as increased lipophilicity caused by chelation, redox properties, generation of reactive oxygen species (ROS), and interactions with cellular biomolecules.^47,48^ Because of this complexity, it is difficult to identify simple SAR trends based on a small set of MIC data.

The analysis of interactions between the drugs and the synthesized compounds demonstrated that the presence of compounds **1**–**3** resulted in a slight decrease in MIC values for rifampicin, isoniazid, and ethambutol in the drug-susceptible strain H37Rv and in the monoresistant strain 3715 (Table 5). However, no reduction in MIC values for isoniazid and ethambutol was observed for the MDR strain 210/21, suggesting limited ability of the compounds to overcome resistance in that strain. Among the tested combinations, the most pronounced MIC reduction was seen with isoniazid in the H_37_R_v_ strain (as low as 0.0161 μg/mL when combined with compound **1**), compared to the MIC of INH alone (0.0625 μg/mL; see Table 4).

**Table 5:** Minimal Inhibitory Concentration Values (MIC) Obtained for Antibiotics Tested in the Presence of Compounds **1**–**3**

	**MIC (μg/mL) for rifampicin in a presence of compounds**		
**comp.**	**H**_**37**_ **R**_**v**_	210/21	**3715**
**1**	0.125	256	0.125
**2**	0.125	256	0.125
**3**	0.125	256	0.125

**Comp.**	**MIC (μg/mL) for isoniazid in a presence of compounds**		
	**H**_**37**_ **R**_**v**_	210/21	**3715**
**1**	0.0161	16	0.0161
**2**	0.031	16	0.25
**3**	0.031	16	0.25

	**MIC (μg/mL) for ethambutol in a presence of compounds**		
**Comp.**	**H**_**37**_ **R**_**v**_	210/21	**3715**
**1**	0.0625	4	0.0625
**2**	0.125	4	0.125
**3**	0.125	4	0.125

The presence of rifampicin led to a noticeable reduction in the MIC values of all three compounds across all strains, from 312.5 μg/mL to 39.06–78.12 μg/mL, suggesting a potential additive or partially synergistic interaction. In contrast, coadministration with INH or EMB did not significantly affect the MIC of compound **1** and had only a moderate MIC-reducing effect on compounds **2** and **3**, particularly in the drug-susceptible strain (Table 6).

**Table 6:** Minimal Inhibitory Concentration Values (MIC) Obtained for Compounds**1**–**3** Tested in the Presence of Antibiotics

	**MIC (μg/mL) for compound 1 in a presence of antibiotics**		
**Drug**	**H**_**37**_ **R**_**v**_	210/21	**3715**
INH	312.5	312.5	312.5
RIF	39.06	78.12	39.06
EMB	312.5	312.5	312.5

	**MIC (μg/mL) for compound 2 in a presence of antibiotics**		
**Drug**	**H**_**37**_ **R**_**v**_	210/21	**3715**
INH	78.12	156.25	156.25
RIF	39.06	78.12	39.06
EMB	156.25	156.25	156.25

	**MIC (μg/mL) for compound 3 in a presence of antibiotics**		
**Drug**	**H**_**37**_ **R**_**v**_	210/21	**3715**
INH	78.12	156.25	156.25
RIF	39.06	78.12	39.06
EMB	156.25	156.25	156.25

Across all tested combinations, no synergistic or antagonistic interactions were observed. Most compound–antibiotic combinations exhibited additive or indifferent effects. For instance, combinations of compound **1** with INH showed additive interactions (FICI = 0.625–0.750), while combinations with RIF and EMB mostly fell within the range of indifference (FICI > 1.0) (Table 7). Similar trends were observed for compounds **2** and **3** with INH. However, the combination of compounds **2** and **3** with RIF and EMB showed additive interactions for the reference strain H37Rv and the monoresistant strain 3715. The results suggest that while compounds **1**–**3** do not enhance the activity of standard drugs synergistically, they also do not interfere antagonistically, making them potential candidates for further structural optimization in combination therapy development.

**Table 7:** Fractional Inhibitory Concentration Indices Obtained for Antibiotics and Combinations of Compounds **1**-**3**

	**FICI values^a^**		
	**H**_**37**_ **R**_**v**_	210/21	**3715**
RIF/1	1.258	2.000	1.016
INH/1	0.625	0.750	0.625
EMB/1	1.125	2.000	1.125
RIF/2	0.746	1.500	0.750
INH/2	0.625	0.750	0.625
EMB/2	0.750	1.500	0.750
RIF/3	0.746	1.500	0.750
INH/3	0.625	0.750	0.625
EMB/3	0.750	1.500	0.750

a FICI value interpretation: synergism ≤ 0.5; additivity >0.5 to ≤ 1; indifference >1 to <4; ≥ 4 – antagonism.

Accordingly, the combination data should be interpreted as evidence of compatibility with standard drugs rather than true synergism. In particular, the observed decreases in MIC values for some combinations are reflected mainly in additive or indifferent FICI ranges and therefore do not justify claims of synergistic enhancement. This more cautious interpretation is consistent with the checkerboard literature, in which reduced MIC values do not automatically imply synergy unless supported by FICI ≤ 0.5.^28,29^

## Conclusions

Three novel copper­(II) complexes with tridentate ONO-donor Schiff base ligands were successfully synthesized and comprehensively characterized using spectroscopic, electrochemical, and crystallographic techniques, complemented by DFT calculations. Theoretical DFT calculations provided valuable insights into the nature of interactions and charge distribution in the studied copper complexes. The energy decomposition analysis revealed that complex **1** exhibits the most stabilizing interactions, dominated by electrostatic and orbital components, whereas in complexes **2** and **3**, these interactions are weakened due to the presence of methanol molecules and increased Pauli repulsion. The charge-transfer and molecular electrostatic potential analyses confirmed a more homogeneous charge distribution in complex **1** compared to those of **2** and **3**. Theoretical analysis of charge distribution and bonding energies complements the spectroscopic and crystallographic data, yielding a coherent picture of how subtle structural differences govern both the catalytic and biological properties. The symmetrical arrangement of counterions in complex **1** further contributes to its enhanced structural stability.

Among the synthesized complexes, dimeric complex **1** demonstrated promising catalytic activity toward the selective oxidation of toluene and styrene under mild conditions, highlighting the potential of Cu­(II)–Schiff base compound in oxidation catalysis. Furthermore, biological assays revealed measurable antimycobacterial effects of all complexes against drug-sensitive and multidrug-resistant *Mycobacterium tuberculosis* strains, while checkerboard studies indicated mainly additive or indifferent interactions with standard anti-TB agents. Overall, these findings underscore the multifunctional nature of Cu­(II)–Schiff base complexes as both efficient oxidation catalysts and biologically active agents. The combined catalytic and therapeutic potential of such systems opens new perspectives for the rational design of transition-metal complexes with dual or synergistic functionalities.

## Supplementary Material



## Notes

Structural tables and additional figures providing MS, PXRD, SEM, EDS, IR, and UV–vis spectra; analysis of electronic structure by DFT calculations (PDF)
