# Supplementary material for: Catalytic and Antitubercular Activities of Multifunctional Copper(II)–Schiff Base Complexes: Insights into Structure, Theoretical Calculations, and Physicochemical Behavior
Source: Inorg Chem. 2026 May 20;65(21):11577–91. doi: 10.1021/acs.inorgchem.5c05547 (PMC13308876; doi:10.1021/acs.inorgchem.5c05547)
Supplement: Supplementary file 1 [file ic5c05547_si_001.pdf]

Supporting Information

Catalytic and Antitubercular Activities of Multifunctional Copper(II)–Schiff Base Complexes: Insights into Structure, Theoretical calculations and Physicochemical Behavior

Anna Jurowska*[1], Weronika Bogdał[1], Janusz Szklarzewicz[1], Mateusz Brela[1], Maciej Hodorowicz[1], Patrycja Miller[2], Mateusz Janeta[2], Agnieszka Głogowska[3], Ewa Augustynowicz-Kopeć[3], Ghodrat Mahmoudi[4,5]

[1] Jagiellonian University, Faculty of Chemistry, Gronostajowa 2, 30-387 Kraków, Poland

[2] University of Wrocław, Faculty of Chemistry, F. Joliot-Curie 14, 50-383 Wrocław, Poland

[3] Department of Microbiology, National Tuberculosis and Lung Diseases Research Institute, Warsaw, Poland

[4] Department of Chemistry, Faculty of Science, University of Maragheh, P.O. Box 55181-83111, Maragheh, Iran

[5]Department of Chemistry, Dogus University, Dudullu-Ümraniye, 34775 Istanbul, Türkiye

*corresponding author e-mail: anna.jurowska@uj.edu.pl

Table of Contents:

S1. The analysis of geometrical parameters from X-Ray measurements. S2

S2. The spectroscopic data (IR-ATR, UV-Vis, stability experiments, SEM, EDS). S5

S3. Analysis of electronic structure by DFT calculations. S16

S1. The analysis of geometrical parameters from X-Ray measurements.

Table S1. Crystal data and structure parameters for **1-3**.

	**1**	**2**	**3**
CCDC	2231666	2232009	2231686
Empirical formula	$C_{36}H_{34}Cl_2Cu_2N_8O_{12}$	$C_{16}H_{17}BrClCuN_3O_7$	$C_{16}H_{15}BrClCuN_3O_6$
Formula weight	968.69	542.22	524.21
Cryst. system	Triclinic	Triclinic	Monoclinic
Space group	$P\bar{1}$	$P\bar{1}$	*C 2/c*
a (Å)	7.5243(3)	9.2118(2)	9.99530(10)
b (Å)	8.9130(3)	10.9441(3)	13.05640(10)
c (Å)	14.9081(4)	10.9484(2)	30.2927(3)
α (°)	104.040(3)	97.368(2)	90
β (°)	94.885(2)	97.107(2)	97.6190(10)
γ (°)	103.109(3)	114.428(2)	90
V ($Å^3$)	934.20(6)	977.37(4)	3918.38(6)
Z	1	2	8
D_{calcd} (mg/m^3)	1.722	1.842	1.777
Absorption coefficient (mm^{-1})	3.394	3.343	5.574
F(000)	494	542	2088
θ range (°)	3.0700 to 76.5990	2.444 to 30.617	5.606 to 77.245
Index ranges	$-8 \leq h \leq 9$<br>$-11 \leq k \leq 11$<br>$-18 \leq l \leq 18$	$-13 \leq h \leq 12$<br>$-14 \leq k \leq 14$<br>$-15 \leq l \leq 15$	$-12 \leq h \leq 9$<br>$-16 \leq k \leq 16$<br>$-38 \leq l \leq 38$
Collected reflections	22542	28652	26991
Independent reflections	3878 [$R_{(int)}$ = 0.0774]	5208 [$R_{(int)}$ = 0.0727]	4117 [$R_{(int)}$ = 0.0491]
Data / restraints / parameters	3878 / 3 / 279	5208 / 0 / 273	4117 / 1 / 258
GOF on F^2	1.079	1.101	1.132
Final R indices [I>2sigma(I)]	R1 = 0.0453,<br>wR2 = 0.1283	R1 = 0.0334,<br>wR2 = 0.0793	R1 = 0.0404,<br>wR2 = 0.1060
R indices (all data)	R1 = 0.0467,<br>wR2 = 0.1298	R1 = 0.0408,<br>wR2 = 0.0818	R1 = 0.0410,<br>wR2 = 0.1064
Residuals (e $Å^{-3}$)	0.769 and -0.892	0.885 and -0.870	0.858 and -0.617

Table S2. Selected bond lengths and angles for **1-3**.

**1**		**2**		**3**	
Bond	Bond length [Å]	Bond	Bond length [Å]	Bond	Bond length [Å]
Cu(1)-N(1)	1.9361(19)	Cu(1)-N(1)	1.9348(17)	Cu(1)-N(1)	1.926(2)
Cu(1)-O(1)	1.9578(15)	Cu(1)-O(1)	1.8828(14)	Cu(1)-O(1)	1.876(2)
Cu(1)-O(1)#1	1.9720(16)	Cu(1)-O(4)	1.9711(15)	Cu(1)-O(3)	1.944(2)
Cu(1)-O(3)	2.2452(16)	Cu(1)-O(3)	2.2432(15)	Cu(1)-O(4)	2.329(2)
Cu(1)-O(2)	1.9770(15)	Cu(1)-O(2)	1.9676(14)	Cu(1)-O(2)	1.953(2)
Cu(1)-Cu(1)#1	3.0128(6)	O(2)-C(8)	1.260(2)	O(2)-C(8)	1.261(4)
O(2)-C(8)	1.264(3)	N(1)-C(7)	1.295(3)	N(1)-C(7)	1.293(4)
N(1)-C(7)	1.284(3)				
**1**		**2**		**3**	
Bond	Angles [°]	Bond	Angles [°]	Bond	Angles [°]
N(1)-Cu(1)-O(1)	91.43(7)	O(1)-Cu(1)-N(1)	92.62(7)	O(1)-Cu(1)-N(1)	94.12(9)
N(1)-Cu(1)-O(1)#1	165.34(7)	O(1)-Cu(1)-O(2)	168.60(6)	O(1)-Cu(1)-O(3)	93.94(9)
O(1)-Cu(1)-O(1)#1	79.89(7)	N(1)-Cu(1)-O(2)	81.27(6)	N(1)-Cu(1)-O(3)	166.23(10)
N(1)-Cu(1)-O(2)	81.44(7)	O(1)-Cu(1)-O(4)	92.57(6)	O(1)-Cu(1)-O(2)	175.39(9)
O(1)-Cu(1)-O(2)	170.84(7)	N(1)-Cu(1)-O(4)	169.51(7)	N(1)-Cu(1)-O(2)	81.47(9)
O(1)#1-Cu(1)-O(2)	105.83(6)	O(2)-Cu(1)-O(4)	92.03(6)	O(3)-Cu(1)-O(2)	90.12(9)
N(1)-Cu(1)-O(3)	95.85(7)	O(1)-Cu(1)-O(3)	96.67(6)	O(1)-Cu(1)-O(4)	92.37(8)
O(1)-Cu(1)-O(3)	96.03(6)	N(1)-Cu(1)-O(3)	96.49(7)	N(1)-Cu(1)-O(4)	92.92(9)
O(1)#1-Cu(1)-O(3)	96.79(6)	O(2)-Cu(1)-O(3)	93.60(6)	O(3)-Cu(1)-O(4)	97.88(9)
O(2)-Cu(1)-O(3)	90.45(6)	O(4)-Cu(1)-O(3)	91.95(6)	O(2)-Cu(1)-O(4)	89.22(8)

#1 -x+2,-y+1,-z+1

Table S3. Hydrogen bonds for **1** [Å and °].

D-H...A	d(D-H)	d(H...A)	d(D...A)	<(DHA)
N(3)-H(3N)...O(6)#2	0.88	2.59	3.340(3)	143.5
N(3)-H(3N)...O(6)#3	0.88	2.52	3.002(3)	115.3
C(2)-H(2)...O(2)#1	0.95	2.18	3.048(3)	151.3
C(7)-H(7)...O(4)	0.95	2.22	3.136(3)	161.1
C(5)-H(5)...O(3)#4	0.95	2.53	3.435(3)	160.0
C(15)-H(15)...N(4)#2	0.95	2.66	3.367(3)	131.3
O(3)-H(3O)...O(4)#5	0.857(9)	1.917(12)	2.720(2)	155(2)
N(2)-H(2N)...O(5)	0.84(3)	1.89(3)	2.723(3)	171(3)
N(2)-H(2N)...N(4)	0.84(3)	2.64(3)	3.429(3)	158(3)

Symmetry transformations used to generate equivalent atoms: #1 -x+2,-y+1,-z+1 #2 x,y-1,z #3 -x+1,-y+1,-z+2 #4 x-1,y,z #5 x+1,y,z

Table S4. Hydrogen bonds for **2** [Å and °].

D-H...A	d(D-H)	d(H...A)	d(D...A)	<(DHA)
O(3)-H(3O)...O(6)#1	0.84	2.45	3.076(2)	131.4
O(3)-H(3O)...O(7)#1	0.84	2.02	2.851(2)	171.0
O(3)-H(3O)...N(3)#1	0.84	2.60	3.394(2)	158.9
C(7)-H(7)...O(5)#2	0.95	2.53	3.469(3)	167.9
O(4)-H(4O)...O(7)	0.87(3)	1.86(3)	2.714(2)	166(3)
N(2)-H(2N)...O(6)#2	0.79(3)	2.00(3)	2.795(2)	174(3)

Symmetry transformations used to generate equivalent atoms: #1 -x+2,-y+2,-z+1 #2 x-1,y-1,z

Table S5. Hydrogen bonds for **3** [Å and °].

D-H...A	d(D-H)	d(H...A)	d(D...A)	<(DHA)
N(2)-H(2N)...O(4)#1	0.88	2.57	3.179(3)	127.1
N(2)-H(2N)...O(5)#1	0.88	2.08	2.954(3)	174.7
N(2)-H(2N)...N(3)#1	0.88	2.69	3.501(3)	154.4
C(7)-H(7)...O(4)#1	0.95	2.34	3.025(3)	128.6
C(7)-H(7)...O(5)#2	0.95	2.63	3.493(3)	150.5
C(3)-H(3)...Br(1)#3	0.95	2.97	3.902(3)	168.3
C(16)-H(16A)...O(1)	0.98	2.42	3.042(4)	120.7
C(16)-H(16B)...O(2)#4	0.98	2.49	3.448(4)	166.9
O(3)-H(3O)...O(4)#4	0.847(10)	2.64(4)	3.295(3)	135(4)
O(3)-H(3O)...O(6)#4	0.847(10)	1.878(14)	2.718(3)	171(5)
O(3)-H(3O)...N(3)#4	0.847(10)	2.55(2)	3.360(3)	160(5)

Symmetry transformations used to generate equivalent atoms:

#1 -x+3/2,-y+3/2,-z+1 #2 x+1/2,y-1/2,z #3 -x+1,y,-z+1/2

#4 -x+1/2,-y+3/2,-z+1

S2. The spectroscopic data (IR-ATR, UV-Vis, stability experiments, SEM, EDS).

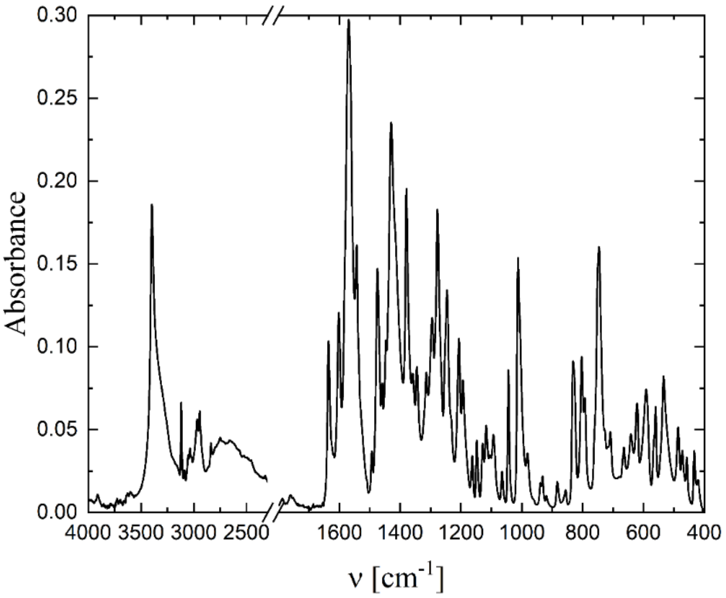

Figure S1. IR-ATR spectrum of **1**.

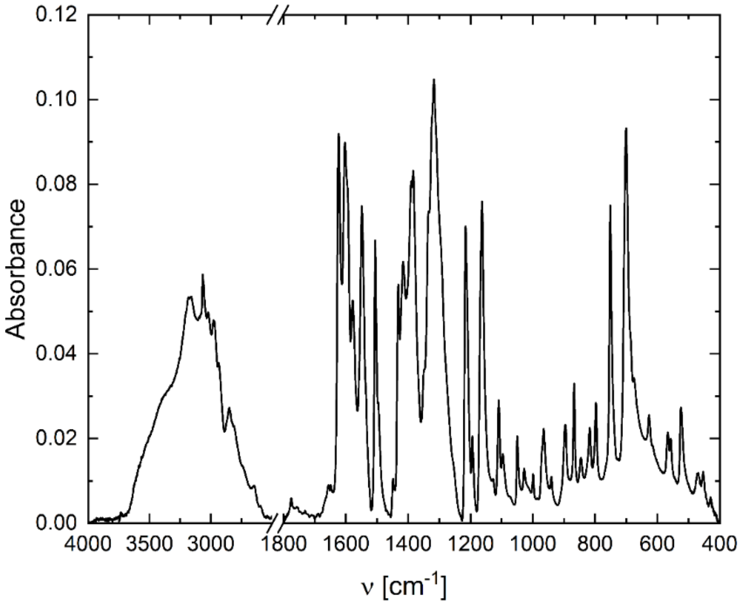

Figure S2. IR-ATR spectrum of **2**.

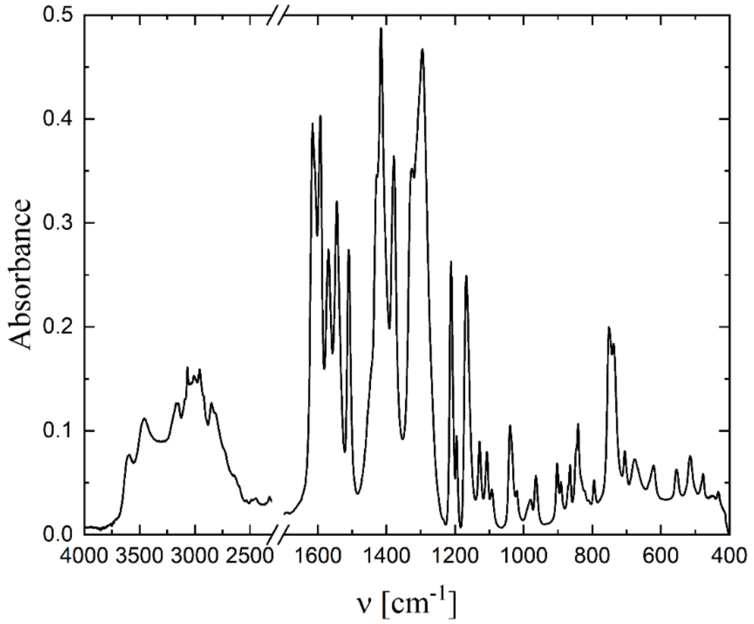

Figure S3. IR-ATR spectrum of **3**.

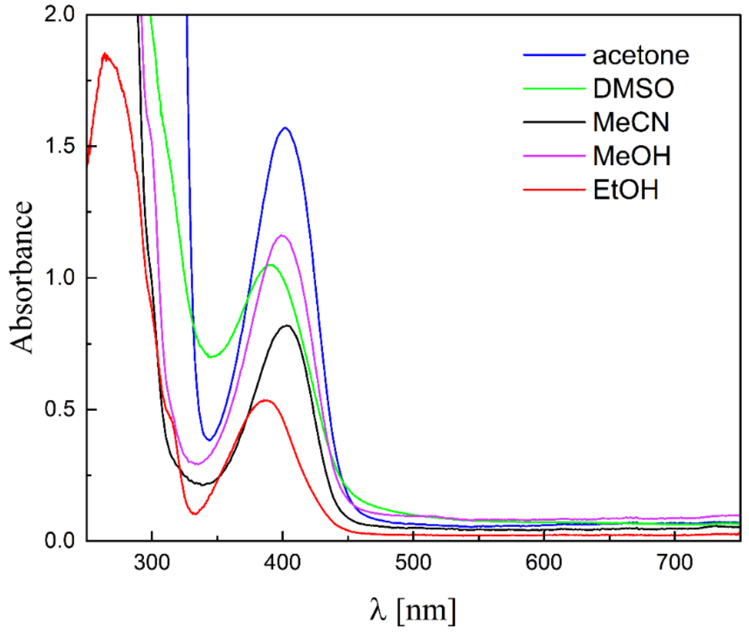

Figure S4. UV-Vis qualitative spectra for **1**.

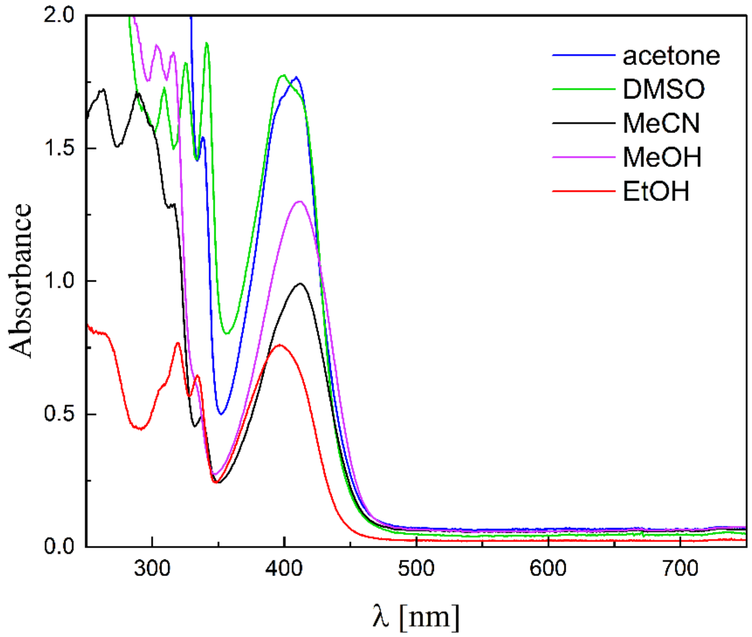

Figure S5. UV-Vis qualitative spectra for **2**.

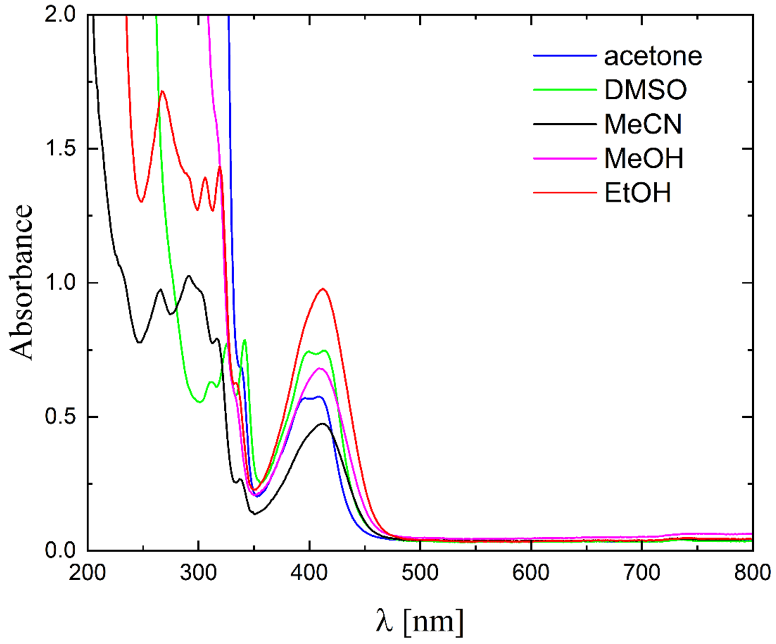

Figure S6. UV-Vis qualitative spectra for **3**.

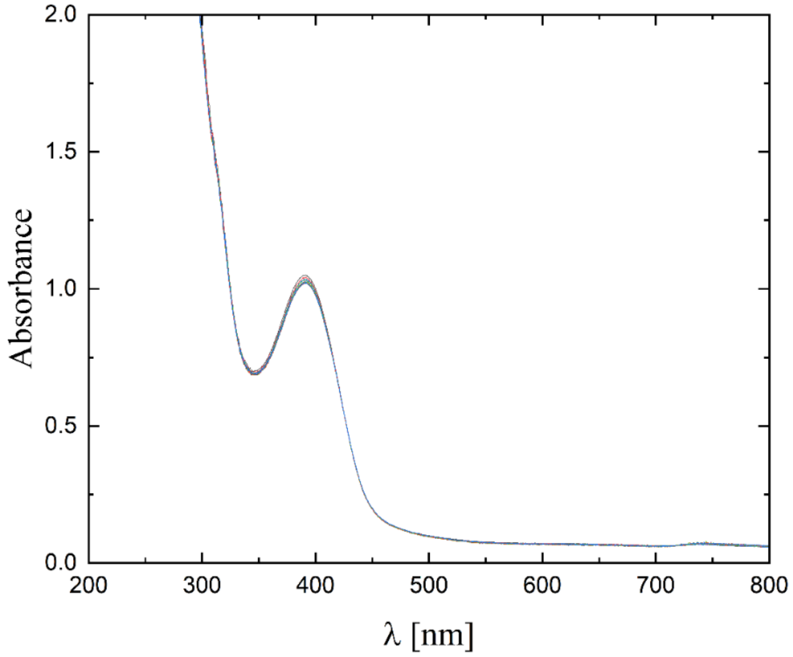

Figure S7. Stability of **1** in DMSO, 15 spectra measured every 420 s.

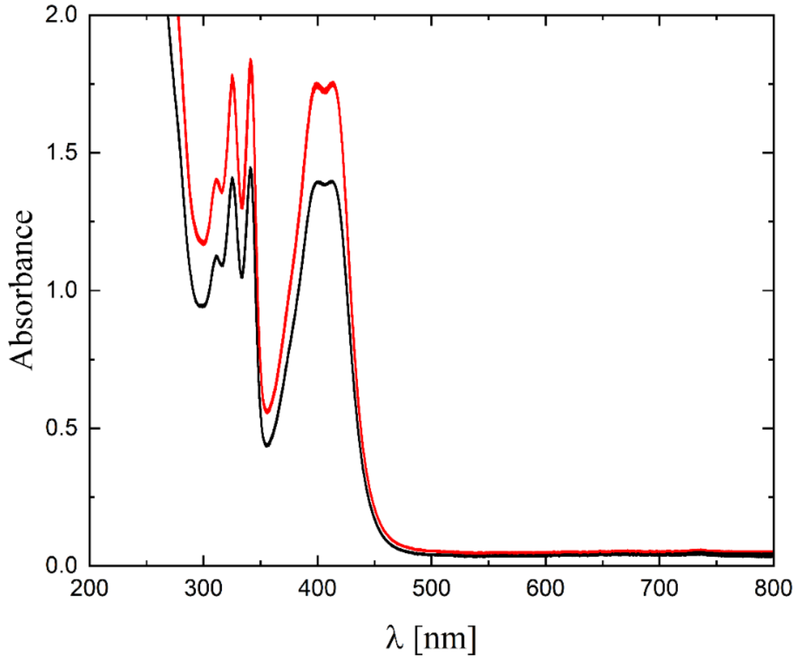

Figure S8. Stability of **2** (black lines) and **3** (red lines) in DMSO, 15 spectra measured every 420 s.

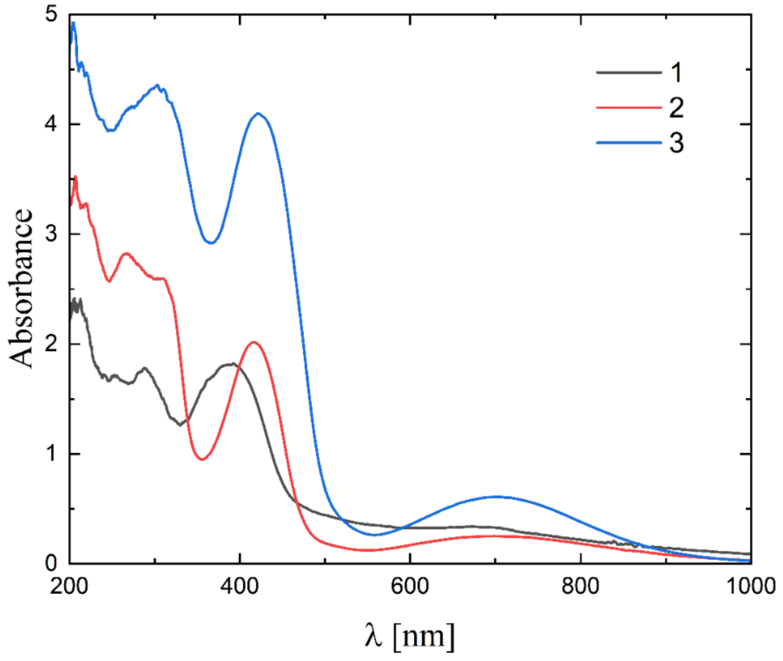

Figure S9. UV-Vis reflectance spectra for **1-3** in $BaSO_4$ after Kubelka-Munk's transformation.

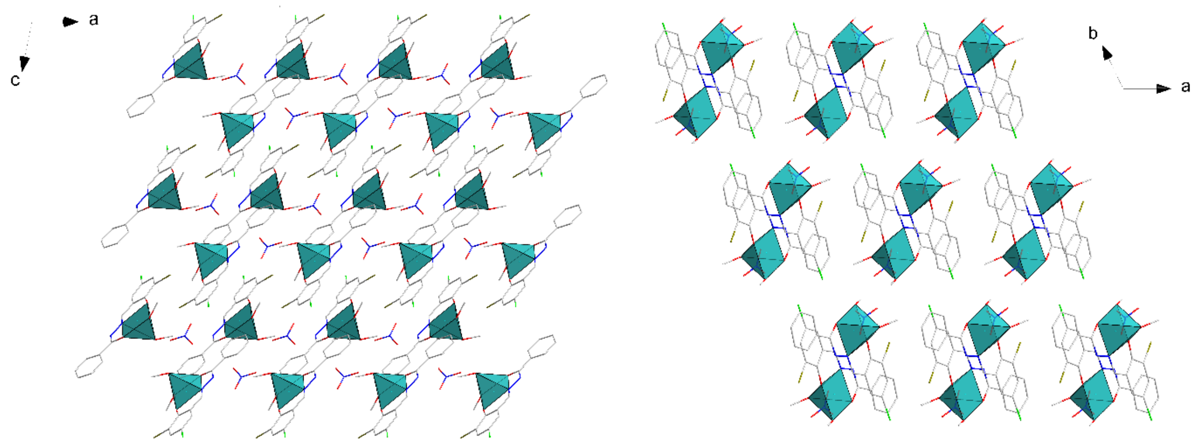

Figure S10. The packing diagram of the crystal structure of **2** viewed along the [010] direction (left side) and [001] (right side).

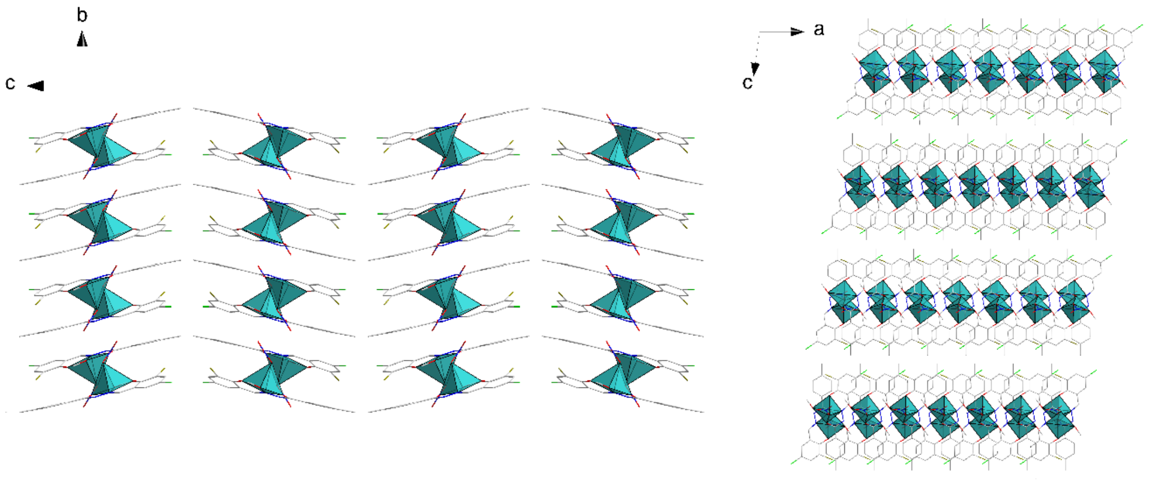

Figure S11. The packing diagram of the crystal structure of **3** viewed along the [100] direction (left side) and [010] (right side).

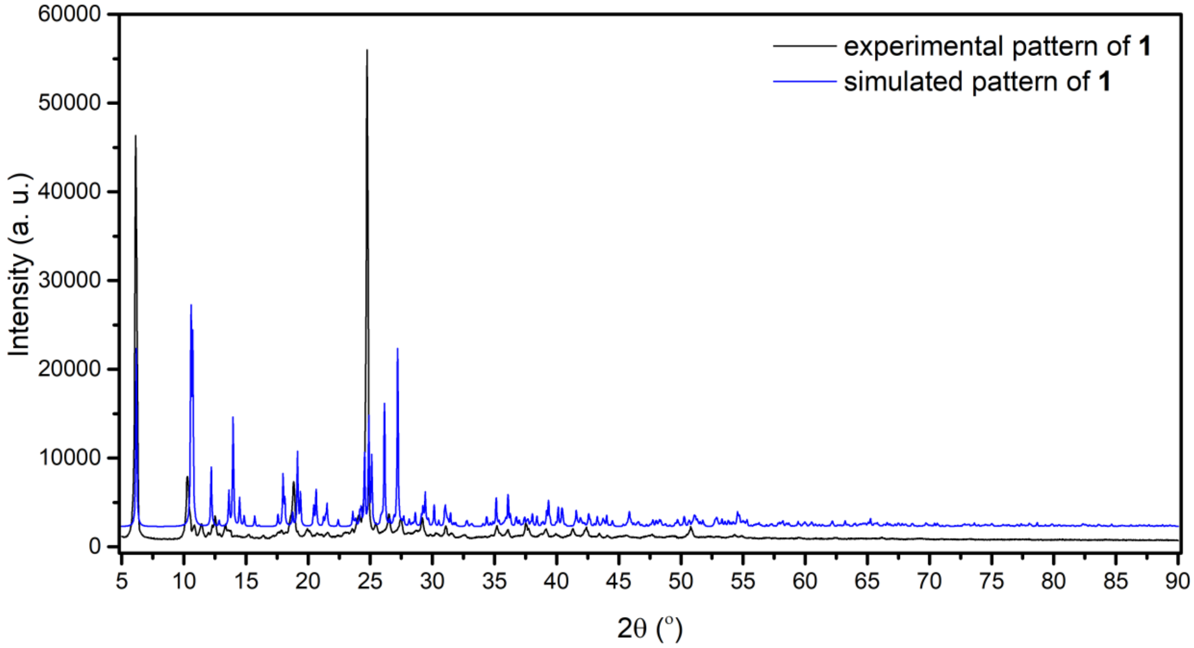

Figure S12. Experimental and simulated pattern of **1**.

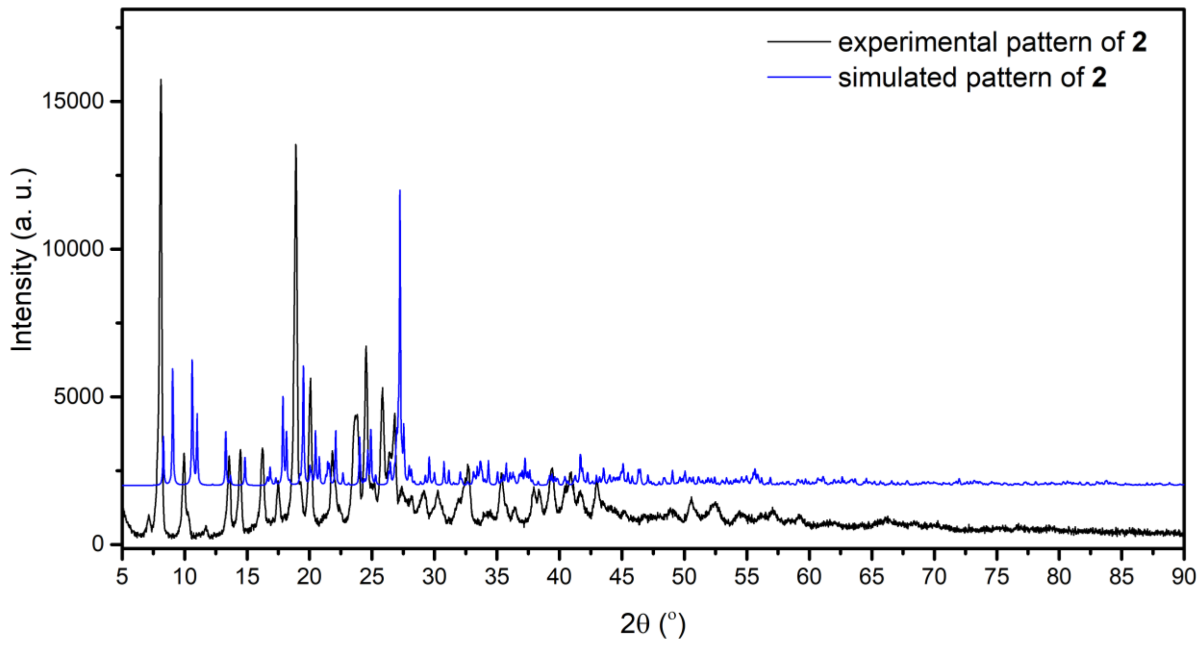

Figure S13. Experimental and simulated pattern of **2**.

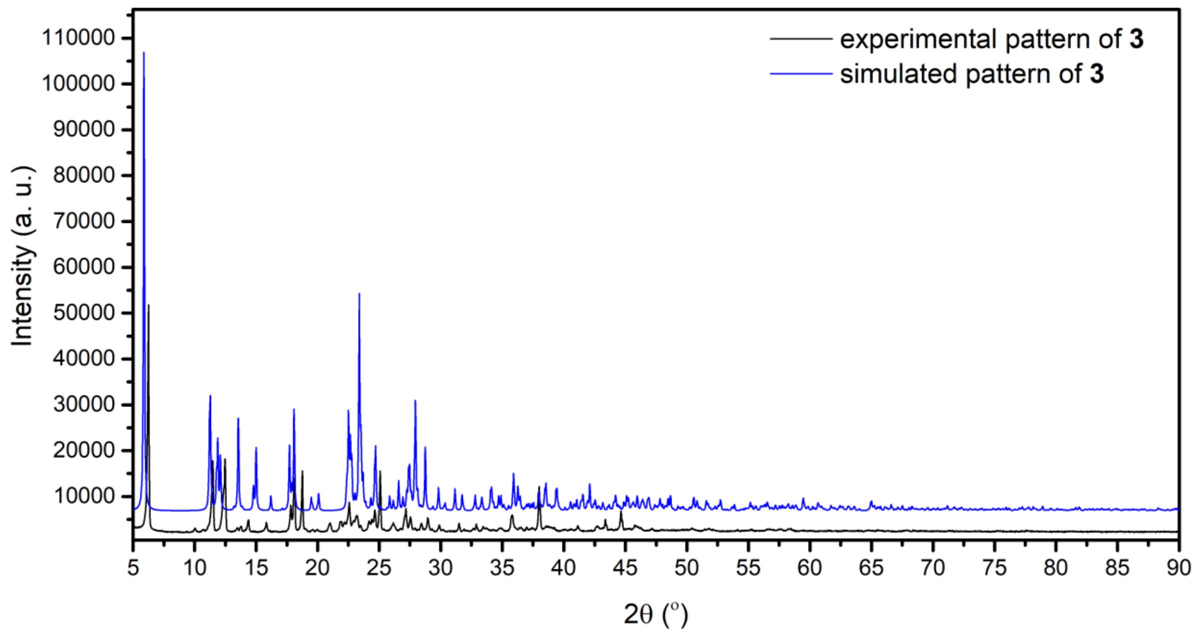

Figure S14. Experimental and simulated pattern of **3**.

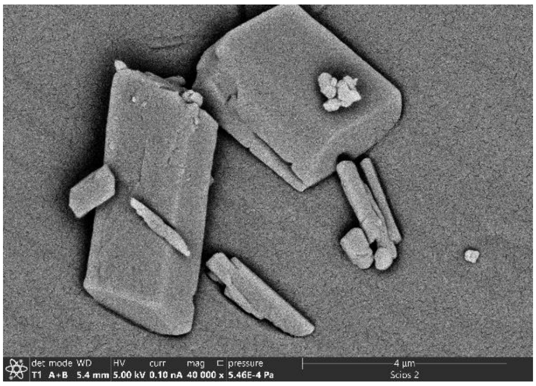

Figure S15. SEM images of **1**.

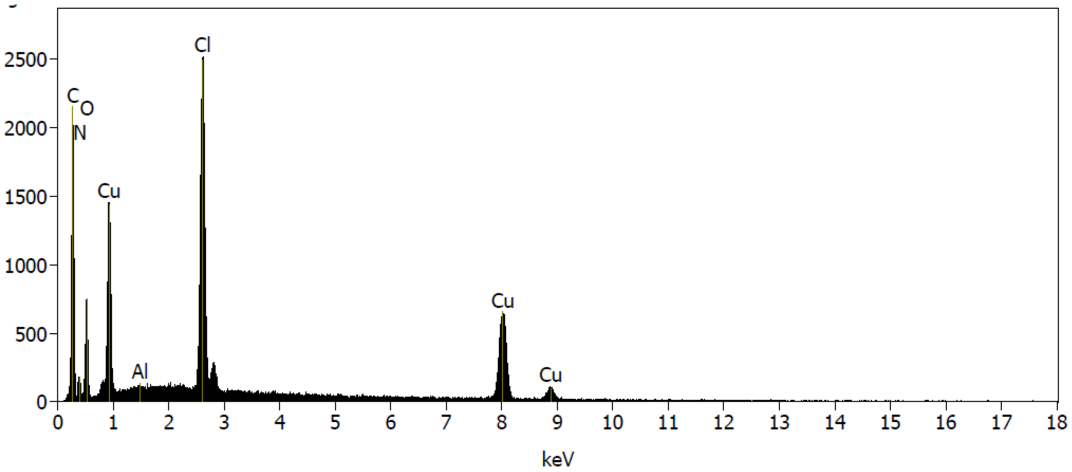

Figure S16. EDS spectrum of **1**.

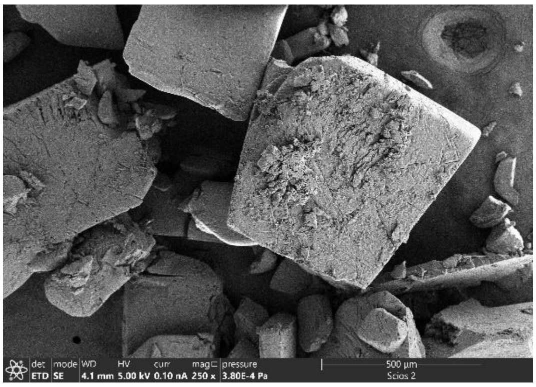

Figure S17. SEM images of **2**.

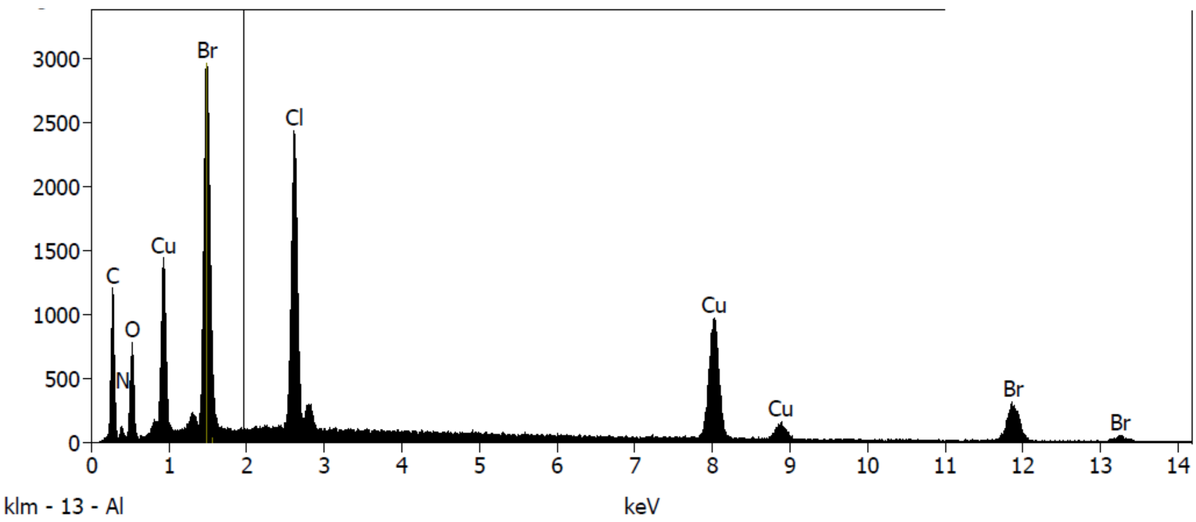

Figure S18. EDS spectrum of **2**.

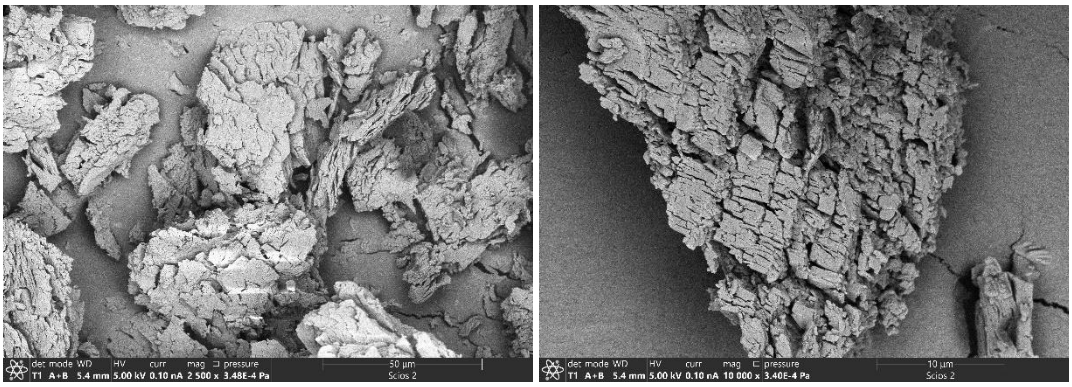

Figure S19. SEM images of **3**.

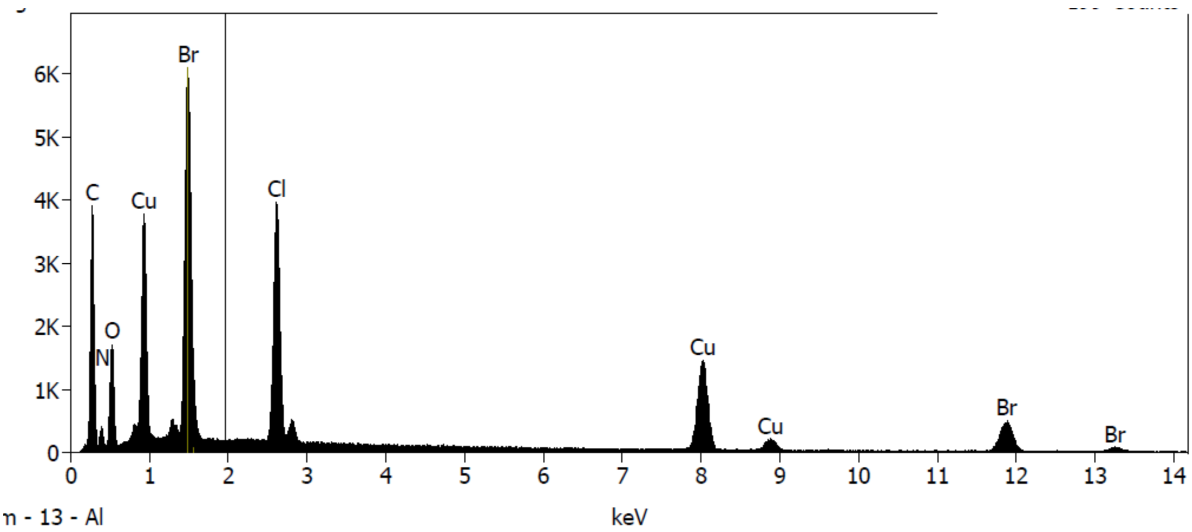

Figure S20. EDS spectrum of **3**.

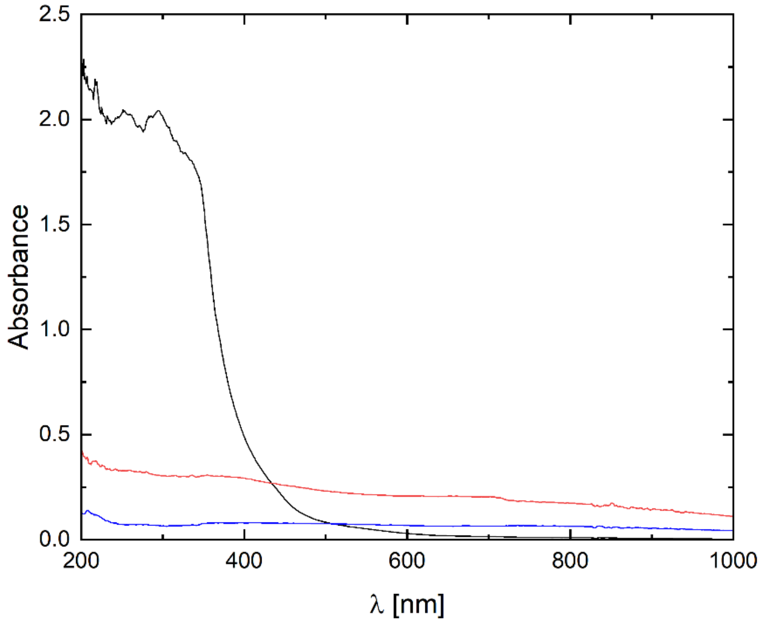

Figure S21. Solid-state UV-Vis diffuse reflectance spectra of **1** after toluene oxidation (blue line), styrene oxidation (red line), and after reaction with H_2O_2 (black line).

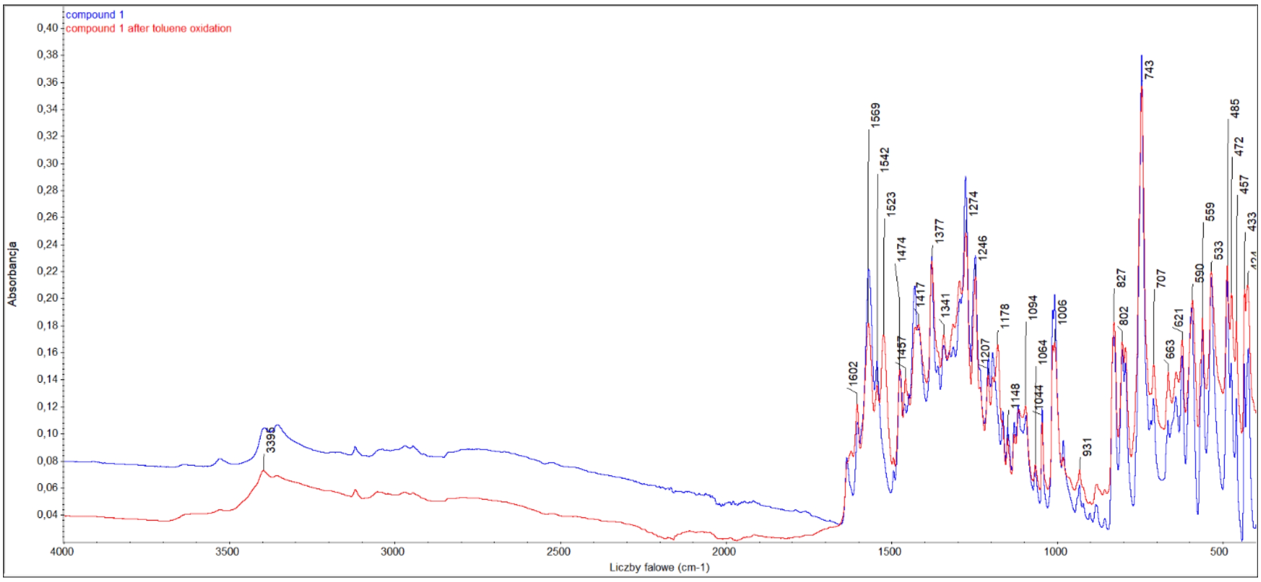

Figure S22. ATR spectra of compound **1** before (blue line) and after (red line) adding H_2O_2.

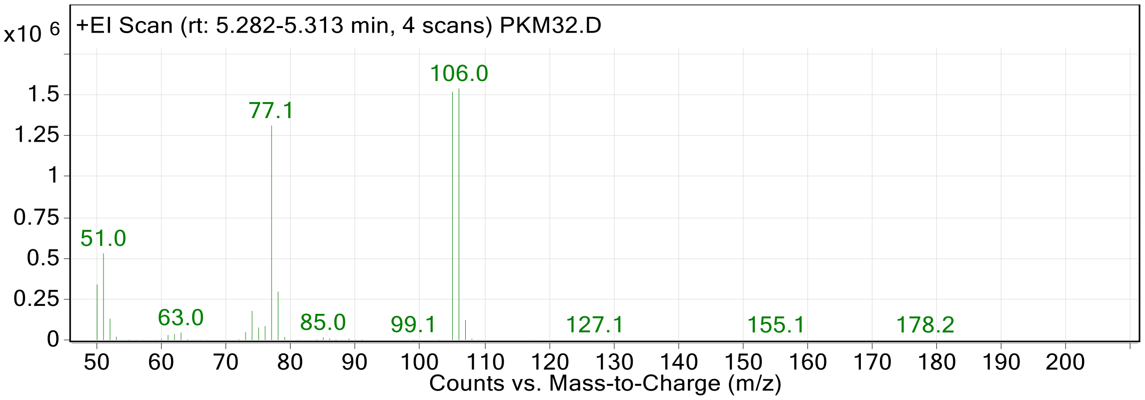

Figure S23. MS (EI) spectrum of obtained benzaldehyde using **1** as a catalyst.

S3. Analysis of electronic structure by DFT calculations.

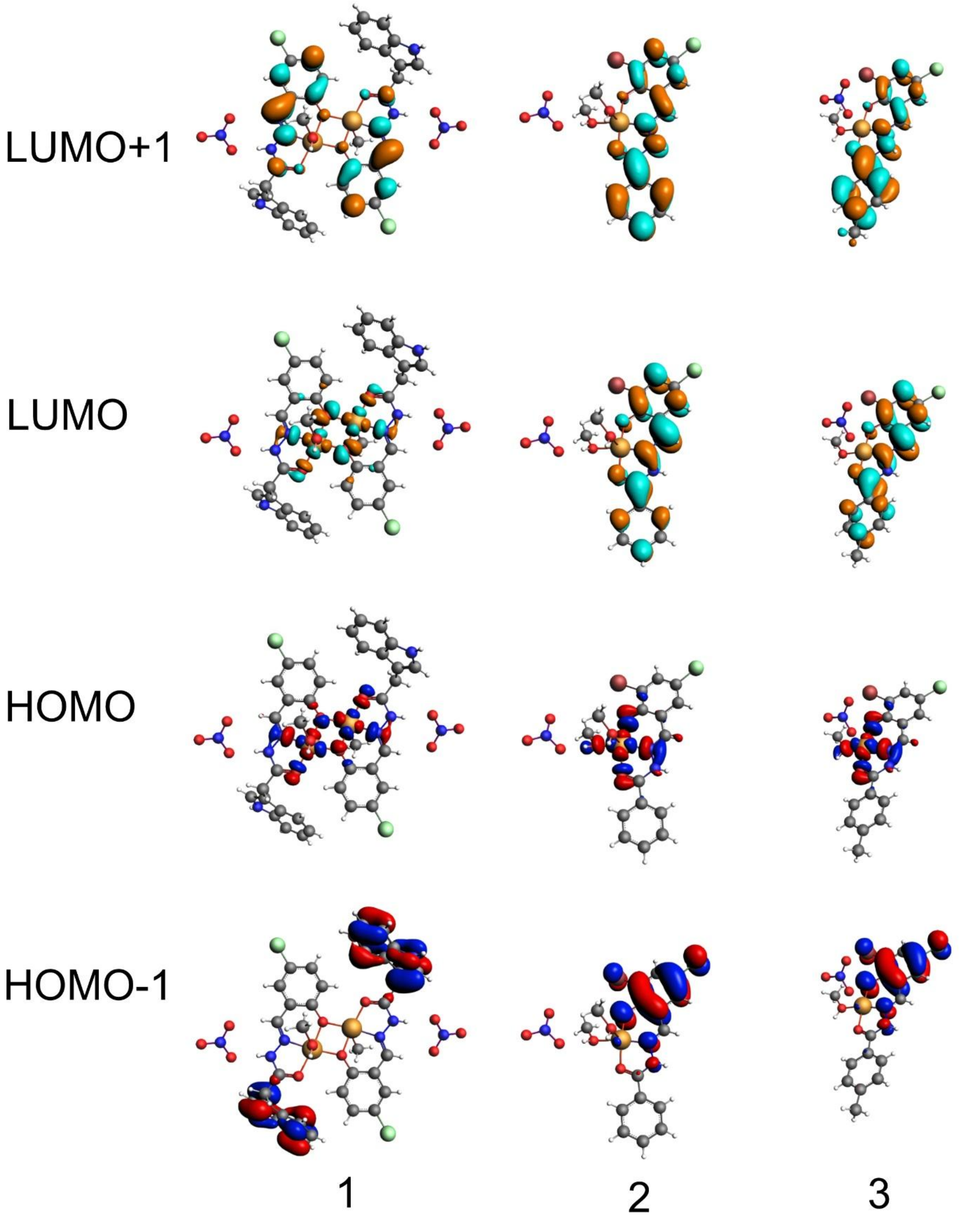

Figure S24. The frontiers molecular orbitals of studied systems **1**, **2** and **3**. The contour values are 0.03 au.

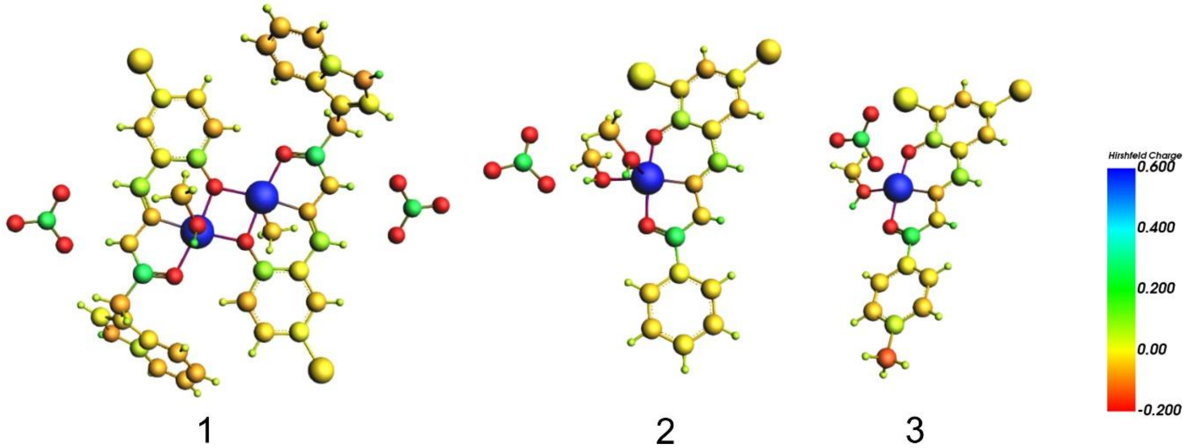

1 2 3

Figure S25. The distribution of Hirschfeld charges of studied systems. The color scale has been show in the right side of Figure.

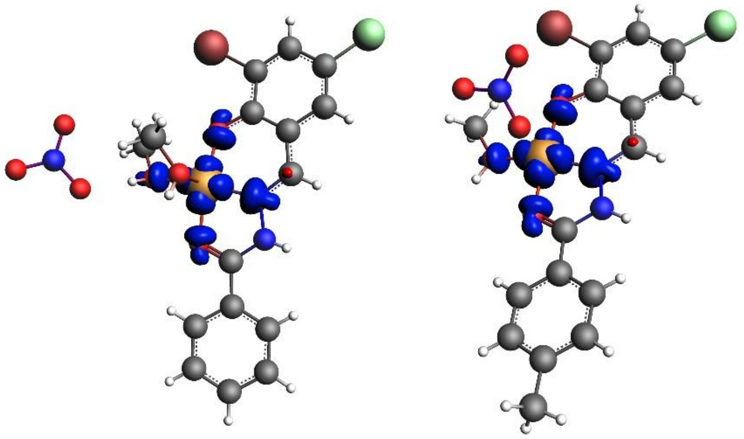

2 3

Figure S26. The spin density differences are calculated by taking the difference between the alpha (α) and beta (β) electron densities. The compound **1** has been omitted because it consists of two copper atoms and the total spin difference is equal to zero.
